# A Bioinspired and Cost‐Effective Device for Minimally Invasive Blood Sampling

**DOI:** 10.1002/advs.202308809

**Published:** 2024-03-07

**Authors:** Nicole Zoratto, David Klein‐Cerrejon, Daniel Gao, Tino Inchiparambil, David Sachs, Zhi Luo, Jean‐Christophe Leroux

**Affiliations:** ^1^ Institute of Pharmaceutical Sciences Department of Chemistry and Applied Biosciences ETH Zurich 8093 Switzerland; ^2^ Institute for Mechanical Systems Department of Mechanical and Process Engineering ETH Zurich 8093 Switzerland; ^3^ Department of Biomedical Engineering Southern University of Science and Technology Shenzhen Guangdong 518055 P.R. China

**Keywords:** bioinspired and cost‐effective device, blood microsampling, concealed microneedles, minimal‐invasiveness, point‐of‐care combination

## Abstract

Conventional venipuncture is invasive and challenging in low and middle‐income countries. Conversely, point‐of‐care devices paired with fingersticks, although less invasive, suffer from high variability and low blood volume collection. Recently approved microsampling devices address some of these issues but remain cost‐prohibitive for resource‐limited settings. In this work, a cost‐effective microsampling device is described for the collection of liquid blood with minimal invasiveness and sufficient volume retrieval for laboratory analyses or immediate point‐of‐care testing. Inspired by the anatomy of sanguivorous leeches, the single‐use device features a storage compartment for blood collection and a microneedle patch hidden within a suction cup. Finite Element Method simulations, corroborated by mechanical analyses, guide the material selection for device fabrication and design optimization. In piglets, the device successfully collects ≈195 µL of blood with minimal invasiveness. Additionally, a tailor‐made lid and adapter enable safe fluid transportation and integration with commercially available point‐of‐care systems for on‐site analyses, respectively. Taken together, the proposed platform holds significant promise for enhancing healthcare in the pediatric population by improving patient compliance and reducing the risk of needlestick injuries through concealed microneedles. Most importantly, given its cost‐effective fabrication, the open‐source microsampling device may have a meaningful impact in resource‐limited healthcare settings.

## Graphical abstract

Bioinspired and cost‐effective device for capillary blood extraction. The device is composed of a storage compartment for liquid blood collection and a minimally invasive microneedle patch hidden within a suction cup. After collection, blood can be safely sent to laboratories for analyses or combined with commercially available point‐of‐care systems for rapid on‐site testing.

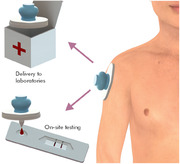

## 1 Introduction

Blood sampling is the basis for ≈70% of medical decisions, being the most prevalent route for disease diagnosis and monitoring.^[^ ^1^, ^2^ ^]^ Conventional venipuncture, although widely practiced, can be invasive and distressing for patients grappling with needle phobia and fear, which affect ≈30% of the general population.^[^ ^3^, ^4^, ^5^ ^]^ This discomfort is especially noticeable in children, where it may result in an increased avoidance of medical treatments involving needles.^[^ ^6^, ^7^, ^8^, ^9^ ^]^ Furthermore, venipuncture is costly as it requires trained personnel, extended sample processing times, and extensive management of biohazardous waste residues.^[^ ^10^ ^]^ These difficulties are even more pronounced in low and middle‐income countries, where healthcare facilities may lack essential equipment, well‐trained specialists, and proper waste disposal systems, leading to increased instances of needlestick injuries (NSIs) and syringe reuse.^[^ ^11^, ^12^, ^13^, ^14^, ^15^ ^]^ Even in developed countries, NSIs remain a significant concern, with an estimated 2 million incidents occurring worldwide each year.^[^ ^16^, ^17^ ^]^ Among them, drawing venous blood is considered the highest‐risk procedure, responsible for 11.5% of NSIs in the US, thereby posing substantial infection risks for healthcare workers and creating additional costs for incident management.^[^ ^18^, ^19^ ^]^

In recent years, the integration of less invasive fingerstick procedures with point‐of‐care (POC) devices has become increasingly popular.^[^ ^10^, ^20^, ^21^, ^22^ ^]^ The latter enables rapid diagnostic tests (e.g., malaria and HIV testing) on‐site in non‐clinical settings, eliminating the need for sample transportation and long analysis times.^[^ ^23^, ^24^ ^]^ Therefore, they can enhance patient compliance while significantly reducing costs and enabling healthcare decentralization.^[^ ^25^, ^26^ ^]^ However, the efficacy of POC devices strongly depends on reliable blood sample methods. In the specific case of fingerstick procedures, high drop‐to‐drop variability can compromise the complete system's accuracy and reliability.^[^ ^27^ ^]^ Furthermore, the typically small volumes of blood provided by fingersticks restrict the applicability of several POC devices or reduce their accuracy.^[^ ^27^, ^28^ ^]^

In an effort to improve current microsampling procedures, alternative technologies have emerged.^[^ ^29^, ^30^, ^31^ ^]^ Haiim (Taiwan) is a vacuum‐assisted blood microsampling device consisting of a lancet and a vacuum component able to collect up to 500 µL of whole blood directly from the fingertips.^[^ ^32^ ^]^ Nevertheless, it requires a trained specialist to oversee the blood collection process.^[^ ^33^ ^]^ More recently, YourBioHealth Inc., (Formerly Seventh Sense Biosystems, USA) developed three touch‐activated phlebotomies (TAP) devices, namely TAP I, TAP Micro (formerly named TAP II), and TAP Micro Select, intended for non‐professionals to collect capillary blood. While recent information on the TAP I device is unavailable, TAP Micro is currently available for investigational use only in the US and is UKCA (UK Conformity Assessed)/CE marked for home use. Finally, TAP Micro Select has received FDA 510(k) clearance with its CE mark pending. Both TAP Micro and TAP Micro Select can withdraw up to 500 µL from the upper arm and effectively address the issue of high result variability associated with fingerstick methods.^[^ ^34^, ^35^ ^]^ Yet, their higher cost compared to venipuncture or fingerstick supplies restricts their use in developed countries. Tasso OnDemand is another blood sampling system from Tasso Inc. (USA) which is currently in development for applications such as at‐home antibody monitoring (TASSO‐SST OnDemand), remote pharmacokinetic monitoring in clinical trials (TASSO‐M20 OnDemand) and anti‐doping testing (TASSO‐SPORT OnDemand).^[^ ^36^, ^37^, ^38^ ^]^ Tasso OnDemand allows collecting up to a maximum of 150 µL of liquid blood directly in an Eppendorf tube or, alternatively, offers a dried blood spot (DBS) sampling kit for dried blood analyses. Loop Medical SA (Switzerland) has recently completed a first‐in‐human study on 100 volunteers with OnFlow, a single‐use device enabling the collection of a larger blood sample (1 mL) directly into a small phlebotomy tube.^[^ ^33^ ^]^ However, both the Tasso OnDemand and OnFlow devices fail to address the needs of low and middle‐income countries due to their high estimated costs (e.g., $25‐30 per unit for Tasso OnDemand). Furthermore, neither of these devices is currently conceived for direct integration with the available POC tests. Considering the limitations of both venipuncture and fingerstick as well as the high cost of the commercially available blood microsampling device, there is still an unmet need for a cost‐effective method for blood collection, especially in low and middle‐income countries.

Here, we describe a cheap, versatile, and single‐use microsampling device for the collection of capillary blood with minimal invasiveness and sufficient volume retrieval for POC tests or laboratory analyses. Inspired by the anatomy of sanguivorous leeches, the device consists of I) a minimally invasive microneedle (MN) patch hidden into a suction cup to enhance patient's acceptance and reduce the risk of NSIs, and II) a storage compartment loaded with an anticoagulant for liquid blood collection. The device geometry, MN array design, and the anticoagulant coating were optimized and their fabrication was adapted to meet the needs of the resource‐limited settings. Computational analysis with Finite Element Method (FEM) was used to predict the device's stress and strain energy distribution profiles during compression and all the computational data were experimentally validated. Finally, an in vivo study on piglets confirmed the device's ability to successfully collect blood and its safety during sample withdrawal. The easy‐to‐use design of the device may enable the collection of capillary blood without the need for healthcare specialists. The collected blood sample may be delivered for laboratory analyses or be combined on‐site with a wide range of commercially available POC systems for real‐time diagnosis and disease monitoring. Additionally, the device's low cost, minimal invasiveness, and combinability with POC systems make it a more accessible and efficient alternative compared to the existing liquid blood microsampling devices. This innovation has the potential to improve healthcare, especially benefiting children and underserved communities in low and middle‐income countries.

## 2 Results and Discussion

### 2.1 Leech‐Inspired Design of the Blood Microsampling Device

**Figure 1:**
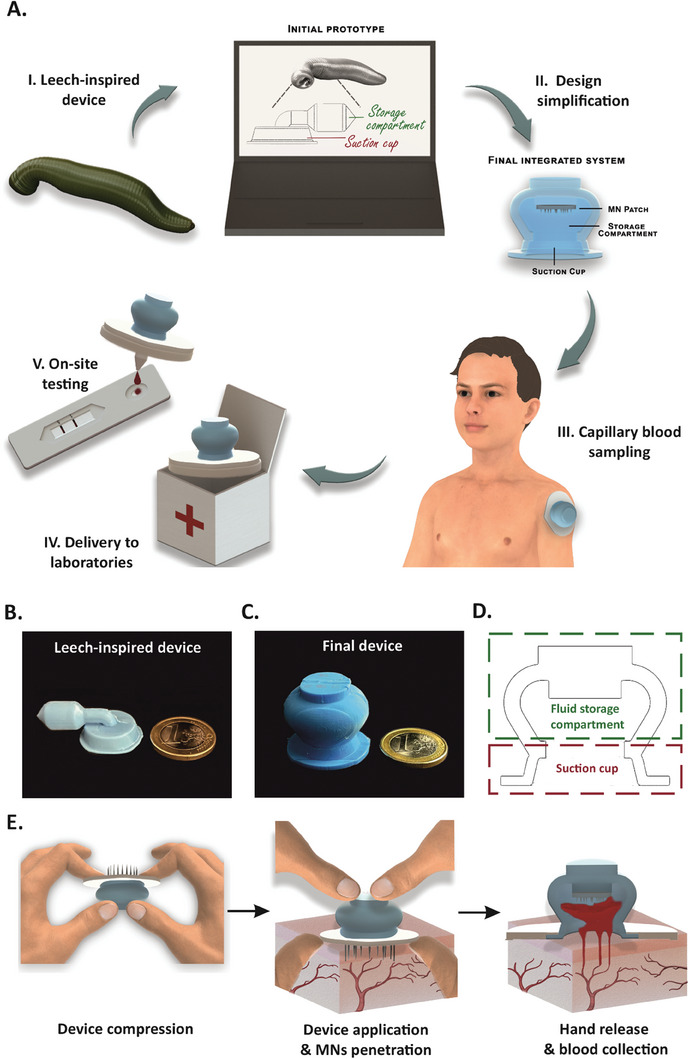
Operation principle of the leech‐inspired blood sampling device. A) Schematic representation of the workflow of the blood microsampling device. Evolution of the leech‐inspired device from I) the initial prototype to II) the final integrated system. III) Optimized blood microsampling device attached to a human arm for capillary blood collection. IV) Blood sample delivery for laboratory analyses. V) Combination of the blood microsampling device with POC systems for on‐site analyses. Photograph of the cast‐molded, B) leech‐inspired, and C) final device close to one‐euro coin for size comparison. D) Sketch of the optimized device, highlighting the different compartments. E) Operating principle of the microsampling device. Compression of the device triggers the deployment of the MNs, creating small punctures into the skin upon attachment. Thereafter, the release of compression force generates a negative pressure that facilitates blood drawn into the device.

The operation principle of the blood sampling device was inspired by the anatomy of sanguivorous leeches (**Figure** **1**A). Similarly to the leech, the blood microsampling device utilized the negative pressure generated by the compression of the reservoir to assist blood withdrawal and storage. Moreover, it incorporated a hidden MN patch to mimic the numerous microteeth present in the leech jaws (Figure 1A,B). The MNs' lower invasiveness, minimal discomfort, and efficient skin penetration should facilitate wound healing when compared to the 2‐mm triangular incision typically generated by leech jaws.^[^ ^39^ ^]^ Additionally, the concealed MN array aimed to mitigate the risk of NSIs resulting from accidental punctures with the MNs.^[^ ^40^ ^]^ Finally, the device had a fluid storage compartment, which could be loaded with anticoagulants similar to the leeches’ salivary secretions, to suit the final application. The original leech‐inspired design was then simplified to enable a more patient‐friendly application (Figure 1A,C,D). The resulting microsampling device is intended to be applied on the upper arm as a previous study showed higher blood flow rates in this location compared to the forearm (Figure 1A).^[^ ^35^ ^]^ After blood collection, the device can be securely sealed and sent to diagnostic laboratories for analyses (Figure 1A) or it can be integrated with POC devices for rapid on‐site testing (Figure 1A).

### 2.2 Device Fabrication and Design Optimization

**Figure 2:**
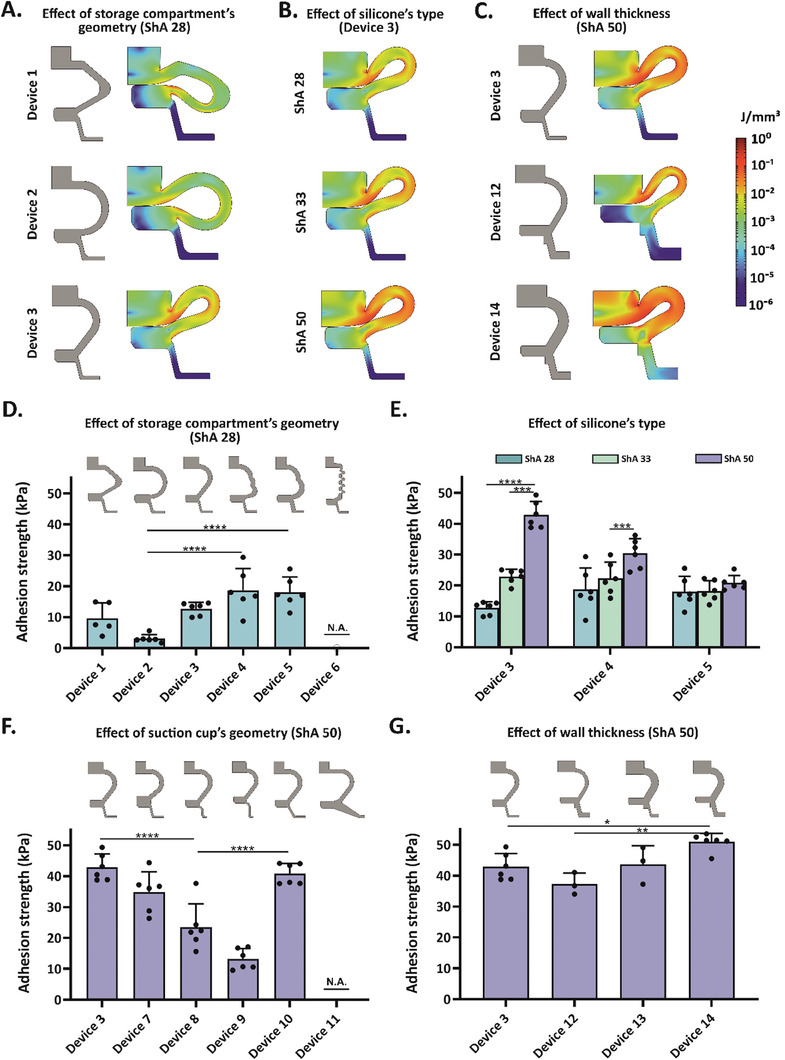
Device design and geometry optimization study. Effect of A) storage chamber's geometry, B) silicone ShA hardness, and C) wall thickness on the strain‐energy distribution profile for different microsampling devices at compression, as assessed by FEM simulation. Maximum compression was deemed at a distance of 0.1 mm between the inner walls of the storage compartment. Effect of D) storage chamber's geometry, E) silicone hardness, F) suction cup's geometry, and G) wall thickness on the adhesion strength of the microsampling devices, as experimentally determined. The study involved manually compressing the devices onto fresh extracted porcine skin and measuring adhesion strength during device pull‐off at a 0° angle. Data are expressed as mean + SD (*n* = 3‐6). Statistical significance was calculated by one‐way analysis (A, C, and D) or two‐way analysis (B) of variance (ANOVA) with Tukey's comparison test with *p < 0.05, **P <0.01, ***p < 0.001 and ****p < 0.0001.

The initial leech‐adapted design required a two‐step application to collect capillary blood (Figure S1A, Supporting Information) and provided a very low adhesion strength (Figure S1B, Supporting Information). Therefore, the device was first simplified to enable a single‐handed operation (devices 1, 2, and 3 in Table S1, Supporting Information). Specifically, a single compression of the storage compartment triggered both the penetration of the MN array into the skin and the generation of negative pressure to initiate blood extraction (Figure 1E).^[^ ^31^ ^]^ Subsequently, FEM analysis was performed solely on the storage compartment of devices 1, 2, and 3 to predict the stress and strain energy distribution profiles at compression, yielding valuable insights into the influence of geometry and material on the stored energy levels and stresses (Figure **2**A and Figure S2A, Supporting Information). Higher levels of stored energy thereby can create larger negative pressures, yet require more force to be applied. Together, these parameters supported the modification of the device shape to enhance ease of application, adhesion strength, and negative pressure generated. The compression of the storage compartment was assumed to be the main contributor to the generation of the negative pressure, due to its larger volume by 4.5 times with respect to the suction cup section (Table S1, Supporting Information). The heart‐shaped design of device 3 yielded a more uniform strain energy distribution with a 3.4 and 3.2‐fold higher average value than the bevel‐triangular (devices 1) and the circular (device 2) ones, respectively (Table S2, Supporting Information). Consequently, the residual air volume in the lateral pockets of device 3 was reduced by 61 and 43%, in comparison to devices 1 and 2, respectively (Figure 2A and Table S2, Supporting Information). As a result, device 3 was predicted to deliver a higher negative pressure, adhesion strength, and blood sampling performance. Furthermore, increasing the silicone shore A (ShA) hardness in device 3 (from 28 to 50 ShA) and its wall thickness (resulting in device 14) led to higher stored energy and thus, improved performance (Figure 2B,C and Figure S2B,C, Supporting Information).

The FEM results were experimentally validated. Various devices differing in the geometry of the fluid storage compartment, silicone material used for fabrication (ShA 28, 33, and 50), design of the suction cup (lower chamber), and wall thickness were tested with respect to their adhesion strength (Figure 2D–G and Table S1, Supporting Information). They were fabricated by mold‐casting with poly(dimethylsiloxane) (PDMS), which was chosen for its good flexibility (Figure S3, Supporting Information). Initially, the impact of the fluid storage compartment geometry on the adhesion strength was investigated (Figure 2D). These devices, fabricated with 28 ShA silicone, shared an identical suction cup design and comparable inner volumes (Table S1, Supporting Information). Their adhesive properties were measured ex vivo on freshly extracted porcine skin by analyzing the pull‐off force with a texture analyzer, except for device 6 since its design precluded vertical compression (Figure S4, Supporting Information). Given that adhesion strength is influenced by several factors, including negative pressure, friction, and sealing, and considering the critical roles of both negative pressure and adhesion to the skin in ensuring the effective operation of the device, these measurements served as a reliable indicator of its overall performance. The heart‐shaped‐based devices 3, 4, and 5 displayed the highest adhesion strengths (18.7 ± 7.0, 12.7 ± 2.0, and 18.0 ± 8.1 kPa, respectively). Consequently, they were selected for further optimization using silicone with different ShA hardness (28, 33, and 50) (Figure 2E). Among the tested materials, ShA 50 silicone was preferred due to its higher stiffness (Young's modulus of 2.0 ± 0.1 MPa), generating enough force to significantly deform the skin while still allowing manual compression (Figure S5, Supporting Information). The improved adhesion strength of device 3 closely aligned with the FEM results. Thereafter, the effect of the suction cup geometry was investigated (Figure 2F). Bottom angles between 103° and 120° (devices 3, 7, and 10) led to the strongest adhesion force. This could be ascribed to a pronounced suction of the tissue in the device, resulting in a notably increased pull‐off force (Figure S6, Supporting Information).^[^ ^41^ ^]^ In contrast, lower (90°) or higher (160°) bottom angles substantially reduced or inhibited the device's adhesion, respectively (Figure 2F and Table S1, Supporting Information). Finally, the best‐performing system, namely device 3, with a bottom angle of 103° and a neck height of 2.78 mm, underwent further optimization in terms of wall thickness (Figure 2G). Among the tested systems, device 14 with an adhesion strength of 51.8 ± 3.5 kPa and a wall thickness of 2.70 and 1.76 mm for the storage compartment and the suction cup, respectively, was selected for the ultimate prototype development. Once again, FEM results strongly aligned with the experimental data. Nevertheless, since further increases in wall thickness or silicone ShA hardness limited the device's displacement under a 30 N compression force (simulating a manual compression force), no additional increments were considered (Figure S7, Supporting Information).

### 2.3 Assessment of Negative Pressure and In Vitro Performance of the Final Device

**Figure 3:**
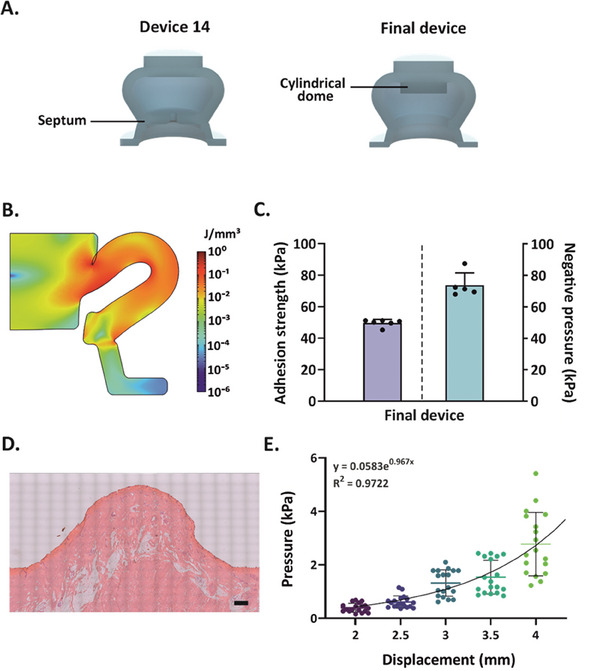
Assessment of the final device's performance. A) 3D cross‐sectional views comparing device 14 (left) and the final device (right). A cylindrical dome was added to the design of the storage compartment, while the silicone separation septum was removed for further MN accommodation. B) Strain‐energy distribution profile of the final device at compression, as assessed by FEM simulation. Maximum compression was deemed at a distance of 0.1 mm between the inner walls of the storage compartment. C) Adhesion strength and negative pressure values of the final device during compression. Experimental data are expressed as mean + SD (*n* = 5–6). D) Skin deformation height, assessed through hematoxylin and eosin (H&E) staining, after the application of the final device. Scale bar: 1 mm. E) Pressure values as a function of skin displacement determined via Nimble tests. The measurements were performed in two independent experiments and each experiment was carried out with nine replicates (*n* = 18).

To house the MN patch within the selected device 14, an inner cylindrical dome (*d =* 12 mm and *h* = 3 mm) was integrated into the fluid storage compartment. Simultaneously, the silicone septum, which originally separated the storage compartment from the suction cup, was eliminated to ensure a complete retraction of the MN patch after skin puncturing (Figure S8, Supporting Information). These modifications led to the final device, featuring an overall height of 2.3 cm and diameters of 1.7 and 2.1 cm for the suction cup and the storage compartment, respectively (Figures 1C,D and 3A). The strain energy distribution profile as well as the adhesion strength of the final device were comparable to that of device 14 (51.8 ± 3.5 kPa and 49.7 ± 2.3 kPa for device 14 and the final device, respectively) (**Figure** **3**B,C and Figure S9, Supporting Information). A similar result was also obtained for the negative pressure (−68.1 ± 1.2 kPa and −73.8 ± 7.9 kPa for device 14 and the final device, respectively), suggesting that the structural modification of device 14 did not affect its performance (Figure S9, Supporting Information). In ex vivo experiments, this negative pressure, generated by the elastic self‐recovery of the silicone device after compression, induced a 5.8‐mm skin deformation (Figure 3D). The relation between the applied pressure and the resulting skin deformation was also determined experimentally (Figure 3E). Results indicated that only 19% (≈14 kPa) of the total pressure accounted for tissue deformation, leaving the remainder available for blood sampling and the enhancement of blood flow around the needle puncture sites.^[^ ^42^ ^]^ Since such a negative pressure value is higher than that of the commercially available TAP device (50‐55 kPa), and a direct correlation between negative pressure and collected blood volume was established, the final device may exhibit better performance in humans.^[^ ^35^ ^]^ Finally, the collection capacity of the final microsampling device was assessed in vitro. Fluid extraction experiments revealed a collected volume of 1603 ± 361 µL, 1450 ± 244 µL, and 793 ± 380 µL for distilled water (DI), blood‐mimicking fluid (BMF), and anticoagulated, fresh porcine whole‐blood, respectively (Figure S10A–C, Supporting Information). The reduced volume of whole blood extracted compared to that of DI and BMF can be ascribed to differences in viscosity. To replicate the complexity of the human skin more accurately, a crosslinked gelatin sponge was introduced into the in vitro setup (Figure S10A, Supporting Information). Also, in this case, the microsampling device was successfully able to sample ≈ 504 ± 262 µL of whole blood.

### 2.4 MN Array Development

**Figure 4:**
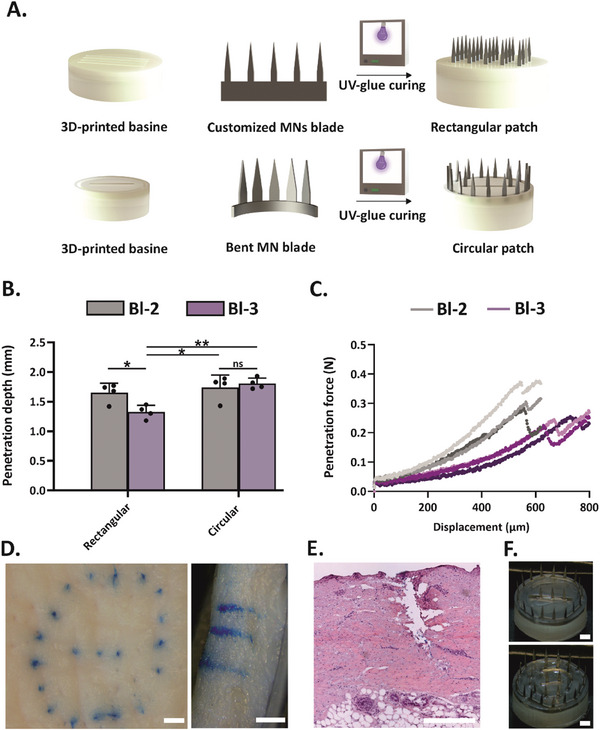
Development and skin penetration performance of the MN array. A) Schematic representation of the MN array development leading to a rectangular or circular patch. B) Penetration depth measurements of circular and rectangular MN arrays composed of Bl‐2 (grey) and Bl‐3 (light purple), as determined ex vivo on porcine ear skin. Data are expressed as mean + SD (*n* = 4). Statistical significance was calculated by two‐way analysis of variance (ANOVA) with Tukey's comparison test with *p < 0.05, **P <0.01. C) Penetration force of a single MN obtained from Bl‐2 (grey) and Bl‐3 (light purple) in porcine ear skin. D) Top (left) and cross‐sectional (right) view of the porcine ear skin after the ex vivo puncturing experiment with a dye‐coated circular patch composed of 4 Bl‐3 (20 MNs in total). Scale bar: 1 mm. E) H&E‐stained section of porcine skin penetrated by the circular MN array composed of 4 Bl‐3 (20 MNs in total). Scale bar: 1 mm. F) Photographs of the circular MN arrays with 4 Bl‐3 (top) and 6 Bl‐3 (bottom), corresponding to 20 and 30 MNs respectively, which were selected for the final version of the device. Scale bar: 1 mm.

MNs have been widely investigated for several applications ranging from transdermal drug delivery to interstitial fluid sampling, thanks to their minimally invasive nature and reduced pain.^[^ ^43^, ^44^ ^]^ In the context of capillary blood sampling, both solid and hollow metal MNs have been employed.^[^ ^35^, ^45^ ^]^ In this work, solid MNs were used due to their ease of manufacturing and superior mechanical properties compared to hollow ones, whilst stainless steel was selected as a fabrication material for its excellent mechanical strength and low manufacturing costs.^[^ ^46^, ^47^ ^]^ Customized MN blades composed of 5 MNs each with an overall needle length and width of 2 mm and 0.35 mm, respectively, were fabricated via laser etching (Table S3, Supporting Information). The 2‐mm needle length was selected to ensure effective capillary access in porcine skin. Nevertheless, for potential human translation, factors such as skin thickness and other anatomical differences between the two species should be considered. Thus, a reduction in MN length from 2 to 1 mm may be required, as in the case of the TAP Micro device. Among the blades, those having a thickness of 75 µm (Bl‐2 and Bl‐3) were selected as a thicker blade (Bl‐1) resulted in limited skin penetration, whereas a thinner one (Bl‐4) easily bent during skin insertion (Figure S11, Supporting Information). The two selected blades, Bl‐2 and Bl‐3, greatly differed in terms of needle pitch and tip angle, with Bl‐3 being sharper (tip angle 13.4°) (Figure S12, Supporting Information). To prepare the MN arrays, the blades were embedded in 3D‐printed basins, whose dimensions were optimized according to the evaluation of skin deformation, as described in section 2.3. (**Figure** **4**A). Specifically, a basin height of 3.0 mm was chosen to accommodate a skin deformation of 5.8 mm, hence enabling complete needle exposure and removal from the stretched skin during and after device application, respectively. Subsequently, dye‐coated, MN arrays with 20 or 30 MNs, in either rectangular or circular configurations, were mounted in the final devices and their penetration depth was evaluated ex vivo (Figure S13, Supporting Information). The circular MN array allowed a deeper MN penetration into the skin than the rectangular one (1.75 ± 0.21 versus 1.65 ± 0.16 mm and 1.81 ± 0.09 versus 1.33 ± 0.11 mm for Bl‐2 and Bl‐3, respectively) (Figure 4B). Although the penetration depth was comparable between the circular MN arrays of Bl‐2 and Bl‐3, penetration force analyses performed on a single MN revealed that Bl‐3 required ≈25% less force for its insertion into the skin compared to Bl‐2 (Figure 4C). Histological assessment further confirmed that the circular array composed of Bl‐3 effectively penetrated the porcine skin's epidermis and reached the deeper dermal layer where capillaries are located (Figure 4E). Consequently, for the final prototype development, a circular array composed of 20 and 30 MNs of Bl‐3 was selected (Figure 4F).

### 2.5 Prototype's Assembling

**Figure 5:**
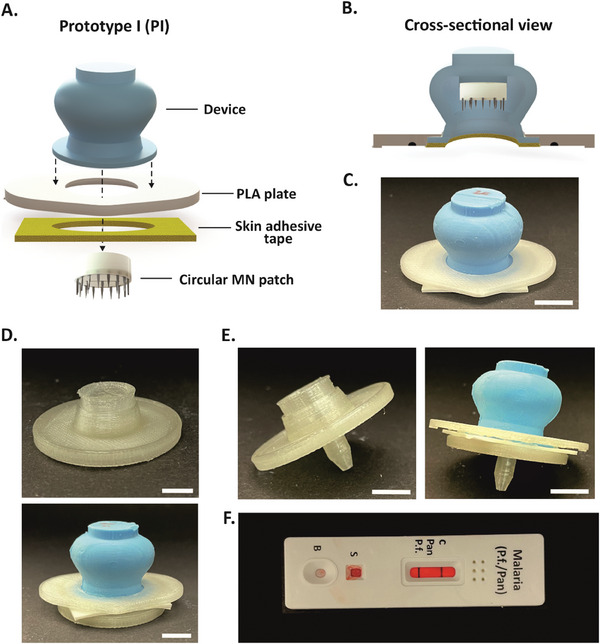
Device assembling. A) Composition scheme of the final prototype (PI). PI features a circular MN patch mounted into a PDMS, cast‐molded device, which was then glued to a 3D‐printed PLA plate and a medical‐grade adhesive to form an air‐tight seal with the skin. B) Cross‐section view of the assembled PI prototype unveiling its inner structure. C) Photographs of the front view of the resulting PI. Scale bar: 1 cm. D) PLA, 3D‐printed lid (top), and its combination with PI (bottom) enable a secure seal for blood transportation to laboratories for analyses. Scale bar: 1 cm. E) PLA, 3D‐printed adapter (left), and its combination with PI (right) to allow integration with POC systems for on‐site analyses. Scale bar: 1 cm. F) Integration of PI with the commercially available POC test for malaria *P. falciparum* detection. The three bands represent the control, PfHRP2, and pan‐pLDH (an aldolase common to all human *P. species*) test lines for *P. falciparum* and pan‐malaria antigens.

For the final version of the device, two distinct prototypes (PI) were assembled (**Figure** **5**). These two PI shared the same device geometry (final device) with differences for the circular MN patches (Table S4, Supporting Information). Specifically, PI‐20 featured an MN array with 4 Bl‐3, corresponding to 20 MNs. PI‐30 was equipped with an MN array composed of 6 Bl‐3, corresponding to 30 MNs (Figure S14, Supporting Information). These arrays were mounted in the fluid storage compartment of the device and specifically glued on its inner dome (Figure 5B). Furthermore, both prototypes were equipped with an identical 3D‐printed poly(lactic acid) (PLA) plate at the base to facilitate their manual compression during application and the exposure of the MN array (Figure 5B,C). The resulting devices exhibited a compact design, characterized by dimensions closely aligned with those of other microsampling devices already on the market.^[^ ^48^ ^]^ In order to avoid coagulation inside the device, 1.5 mg of the anticoagulant potassium ethylenediaminetetraacetic (K_2_‐EDTA) was added to the storage compartment, an amount sufficient for 1 mL of whole blood. After assembling, the two prototypes were sterilized via autoclaving, since this process had no impact on the device's adhesion strength (Figure S15, Supporting Information). Lastly, the prototypes were equipped with medical‐grade adhesive to enable secure sealing on the uneven and rough skin surface during blood sampling (Figure 5A and Figure S14, Supporting Information). Upon application, the prototypes can be effortlessly detached from the skin by peeling the tape off from one of its top corners (Figure S16, Supporting Information). The blood microsampling devices would be used to deliver blood to laboratories for analyses or be integrated with commercially available POC systems for real‐time analyses. Therefore, a prototype 3D‐printed, PLA lid was developed to facilitate the device's recapping after blood withdrawal, ensuring its safe shipping to a laboratory while preventing sample evaporation or drying and maintaining stable pH levels (Figure 5D, Figure S17 and Video S1, Supporting Information). Additionally, a tailored adapter was fabricated to connect to the suction cup, enabling the elution of a small sample volume when using POC devices (Figure 5E and Video S2). As a proof of concept, 500 µL of human blood (a volume equal to that sampled by the device in vitro) spiked with recombinant Plasmodium falciparum histidine‐rich protein 2 (PfHRP2), a biomarker for malaria infection (LOD = 8 ng/mL), was loaded into the storage compartment of the final device, followed by the elution of a small volume onto a commercial malaria paper‐based test (Figure 5F). It is worth noting that, although several components of the PI were manufactured by 3D printing, their production could be readily translated to cost‐effective injection molding with various thermoplastics. Consequently, through a simple fabrication process employing reusable alumina molds and etching masks for the device and MNs fabrication, respectively, and the use of inexpensive materials (i.e., silicone and stainless‐steel MN blades), the final cost of each microsampling device could be significantly reduced. This cost‐effectiveness could make the devices considerably more affordable compared to other commercially available options, such as Tasso OnDemand, which is priced at $25‐30 per unit.

### 2.6 In Vivo Studies on Piglets

**Figure 6:**
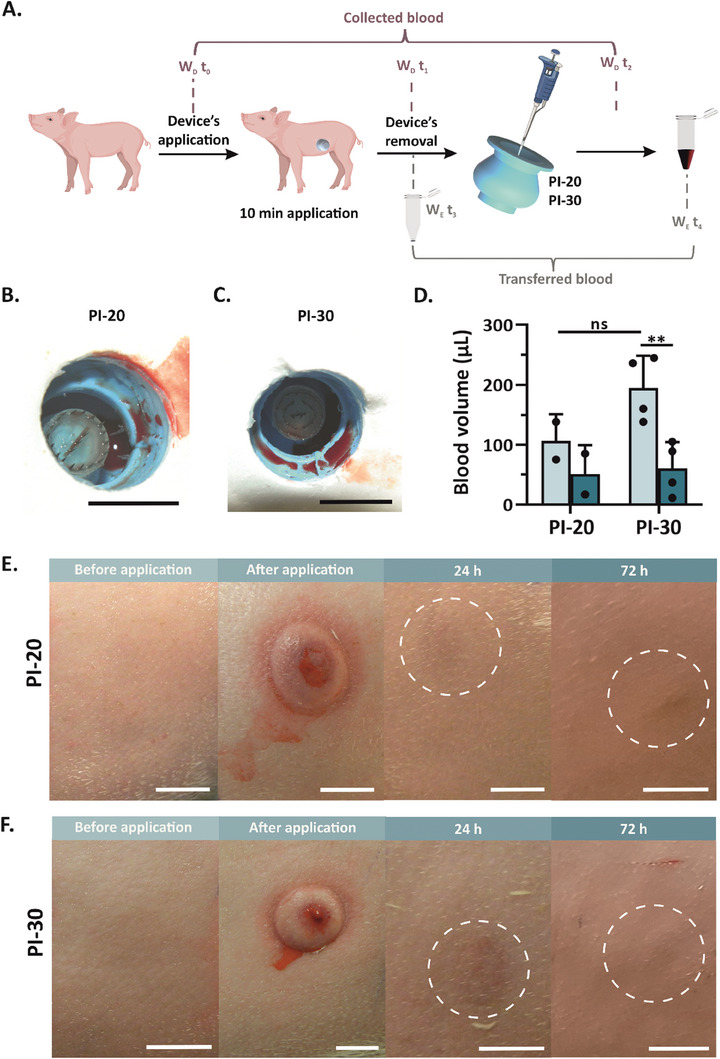
In vivo blood sampling utilizing the microsampling device. A) Workflow of the blood collection process. The prototype's weight was measured prior to application (W_D_t_0_), after removal (W_D_t_1_), and after blood transfer (W_D_t_2_). After collection, the blood was transferred into an Eppendorf tube, with the tube's weight measured before (W_E_t_3_) and after (W_E_t_4_) the transfer step. B) Photographs of PI featuring 20 MNs (PI‐20) and C) 30 MNs (PI‐30) after 10 min application. The collected blood is clearly visible in the storage compartment. Scale bar: 1 cm. D) Blood volume extracted from the piglet's flank after 10 min application (light green), as determined by weighing the device after blood collection, after blood transfer, and after its transfer to an Eppendorf tube (teal) for both PII‐20 and PII‐30. The experiments were performed in two animal models, each with one (PI‐20, *n* = 2) or two (PI‐30, *n* = 4) replicates, respectively. Statistical significance was calculated by unpaired t‐test with a two‐tailed distribution and unequal variance with **p < 0.01 and ns: P > 0.05. (E‐F.) Images of the piglet's skin prior to application, immediately post‐application, at 24 h, and at 72 h after application. The application site is indicated with a dashed white circle. Scale bar: 1 cm.

The performance of the blood sampling devices was evaluated in piglets as their skin thickness is similar to that of humans.^[^ ^49^, ^50^ ^]^ To ensure proper application, the prototypes were compressed by pressing the device's knob until the MNs were fully exposed (Figure S18, Supporting Information). Subsequently, the prototypes were applied to the piglet's flank. After 10 min of application, the devices were removed and the amount of collected blood was determined by weight measurements (**Figure** **6**A). Notably, all prototypes effectively collected whole blood from the piglet's flank (100%) (Figure 6B,C and Figure S19, Supporting Information). Specifically, 107 ± 45 and 195 ± 54 µL of capillary blood were extracted with PI‐20 and PI‐30, respectively. Such a volume is already compatible with many POC instruments.^[^ ^51^ ^]^ Additionally, as the porcine blood viscosity is higher compared to that of humans and pigs are hypercoagulable (they exhibited shorter clot initiation time and reduced clot formation time), we believe that the sampled blood volume would be higher in humans.^[^ ^52^, ^53^ ^]^ Furthermore, the possibility to vary the volume of the storage compartment, the number of MNs, and the silicone ShA hardness may enable the fabrication of a wide range of devices with tunable collection volume capabilities. Thereafter, the collected blood was transferred into an Eppendorf tube, resulting in 51 ± 48 and 61 ± 11 µL of transferred anticoagulated whole blood (Figure 6D and Figure S20, Supporting Information). The lower volume of collected blood compared to the extracted one may be attributed to handling issues and dead volume in the pipette tips. Among the tested devices, the majority successfully drew the collected blood into the fluid storage compartment. Nevertheless, a small amount of the extracted blood remained in the suction cup. This may originate from a sub‐optimal positioning of these devices (most of them were applied to the lateral lane of the flank instead of the central lane) or shaking during device removal (Figure 6B,C and Figure S19, Supporting Information).

To evaluate the safety and the minimal invasiveness of the blood microsampling device, the piglet's skin was systematically photographed during the experiments (Figure 6E,F and Figure S21, Supporting Information). For both prototypes, the application site was clearly visible immediately after the device's removal due to skin deformation and bleeding. The deformation on the piglet's skin originated from the negative pressure generated by the device, which enabled the effective blood drawn from the tissue. Remarkably, 24 h after the removal, no visible wounds attributable to MN penetration were observed, confirming the minimal invasiveness of the blood microsampling prototypes. Only a faint bruise was noticed at the application site, similar to lancet‐based devices.^[^ ^54^ ^]^

## 3 Conclusion

This work aimed to develop an affordable and minimally invasive blood sampling device for use in low‐income countries, improving diagnostics and patient compliance, especially among children. Inspired by the anatomy of sanguivorous leeches, the device consists of a suction cup equipped with a metal MN patch array and a storage compartment containing K_2_‐EDTA as an anticoagulant for liquid blood collection. Combining mechanical analyses with FEM simulation, the device was optimized toward a simple single‐step application and sufficient blood retrieval for laboratory analyses or in‐situ testing. Preliminary in vivo tests in piglets confirmed the ability of the device to extract ≈195 µL of whole blood in 10 min, a volume comparable to the best commercial microsampling devices. Acknowledging that blood coagulation typically begins within 5 min, in further development stages the application time will be reduced to a maximum of 5 min, aligning with both Tasso and TAP devices. After collection, the device can be safely sealed and transported to laboratories for analyses or combined with commercial POC instruments for on‐site diagnostics through a customized adapter, which allows the elution of a small blood volume from the storage compartment. In this respect, the device compatibility with POC tests was successfully confirmed with a spiked PfHRP2 human blood sample, drop‐cast on a malaria test strip. In contrast to commercial devices, e.g. TAP I, our prototype eliminates the need for a pre‐evacuated chamber as the negative pressure is generated by the elastic self‐recovery of the silicone after compression. Additionally, the absence of microfluidic elements, such as channels for anticoagulant coating, could significantly simplify the production process. Overall, owing to its user‐friendly design, cost‐effectiveness, and versatility, our device may be a platform to improve the accessibility of reliable blood diagnostic services in low and middle‐income countries and accelerate the decentralization of healthcare. Finally, thanks to the presence of hidden MNs this device may help to reduce anxiety in children, increase patient compliance, and reduce NSIs.

## 4 Experimental Section

### Materials

Phosphate‐buffered saline pH 7.4 tablets (PBS), 2‐propanol (isopropanol) (≥99.8%), acetone (≥99.8%), methanol‐free paraformaldehyde (PFA), gelatin from porcine skin type A and potassium iodide were purchased from Sigma‐Aldrich. Dulbecco's modified Eagle's medium (DMEM) Nutrition Mixture (with glutamine and 15 mM Hepes, without phenol red) (DMEM/F12) and antibiotic‐antimycotic 100X (10 000 U of penicillin, 10 mg of streptomycin, and 25 µg of amphotericin B per mL) were purchased from Thermo Fisher Scientific. ProLong Diamond Antifade Mountant was purchased from Invitrogen. K_2_‐EDTA, extra pure was from Fisher Chemicals. Glycerol 99% and glutaraldehyde 25% aqueous solution were purchased from ABCR (abcr GmbH). PDMS Bluesil RTV 3428 (ShA 28) was purchased from Elkem. Smooth‐Sil 950 platinum cure silicone (ShA 50) was purchased from Smooth‐On. RTV2 platinum silicones with ShA hardness of 13 and 33 were purchased from Silikon Fabrik. Silbione HCRA 4500 silicone (ShA 70) was purchased from Elkem. Optimal cutting medium (OCT) was purchased from Leica Microsystems. Hydrochloric acid was purchased from VWR. Hematoxylin and eosin staining (Mayers hematoxylin, eosin solution, ethanol (EtOH), PBS, and xylene) were purchased from Wuhan Servicebio Technology Co., Ltd. Recombinant PfHRP2 was purchased from Abcam. Cleartest for malaria *P. falciparum/Pan* detection was purchased from Mein Arztbedarf GmbH. Human whole blood treated with K_2_‐EDTA (pooled, mixed gender) was purchased from BioIVT LLC. Blood samples were collected under ISO, FDA, USDA, and EPA certificates. Customized stainless‐steel blades (Bl‐1, Bl‐2, Bl‐3, and Bl‐4) were fabricated via laser etching and purchased from Wist EU.

### Design of Molds and Stamps For Device's Fabrication

Each 3D device, designed in SolidWorks (Dassault Systemes SE), was inverted to a negative casting mold and stamp, which were then multiplied (2 × 2), enabling the fabrication of 4 devices simultaneously (Figure S3A, Supporting Information). The molds were exported as.STEP files and provided with 4 threaded holes (*d =* 4 mm) using Fusion Autodesk 360. Subsequently, both molds and stamps were exported as.STL files and 3D‐printed via LCD printing (Jenny Light 1+) with a Value Line UV/DLP Tough (ABS‐like) clear resin. After printing, both parts were washed twice with isopropyl alcohol in a sonicator bath at 40 °C for 30 min, followed by a quick rinse with acetone. They were then UV‐cured for several hours (UVP CL‐1000 Ultraviolet Crosslinker). Before being employed in the silicone casting process, molds and stamps were exposed to sunlight for ≈10 days to ensure that no residual radicals from the printing process remained on their surfaces.

### Tensile Test on Commercial Silicones

Tensile tests were performed on a dog‐bone specimen (ASTM 638 type IV) with a gauge length of 13 mm, at a rate of 1 mm min^−1^ (resulting in a stretch rate of 0.0012 s^−1^) using a TA.3DXTplus texture analyzer (Stable Micro Systems) with a 50 N load cell. The specimens were prepared via cast‐molding using an LCD 3D‐printed mold and stamp (Jenny Light 1+). Specifically, a mixture of PDMS base and curing agent was prepared at a ratio of 10 to 1 (w/w) for ShA 28 and 50 silicone and at a ratio of 1 to 1 (w/w) for ShA 33 silicone. After a homogeneous mixing, the PDMS pre‐polymer was degassed under vacuum for 10 min, poured into the mold, and vacuum‐degassed for a further 5 min. Subsequently, the stamp was inserted and the resulting merged plates tightened together in a custom‐made, 3D‐printed holder (Pro 2, Raise 3D Technologies, Inc.) made of PLA (Spectrum filament PLA tough natural, 1.75 mm) with 4 bolts and nuts to uniformly apply pressure during the overnight PDMS hardening. Afterward, the stamp was removed and any excess material was trimmed away. Young's modulus was calculated as the slope of the initial part (first 10 points) of the stress–strain curve.

### Device Deformation Modeling

All the device geometries were designed in Solidworks (Dassault Systemes SE) and subsequently imported into COMSOL Multiphysics 6.1 (COMSOL AB) and converted to a two‐dimensional (2D) cross‐section geometry, benefitting from their rotational symmetry. The Young's Moduli of various silicon materials were experimentally determined by tensile tests (as previously described) and then used as material parameters in the simulation. A Poisson's ratio of 0.49 was assumed in line with literature values for silicone.^[^ ^55^ ^]^ An isotropic and compressible hyper‐elastic neo‐Hookean material model with a Simo‐Pister volumetric strain energy was selected. For the compression simulation, a two‐platform approach was implemented (Figure S22, Supporting Information). Specifically, the lower platform was designated to maintain a predefined zero deformation, while the upper platform was responsible for the application of the compression force. Devices were meshed with triangular elements (Figure S23 and Table S5, Supporting Information). Since the model can be assumed quasistatic, the deformation speed does not influence the model results. Therefore, a very slow deformation rate was chosen to improve convergence of possible contact during deformation, leading to a total simulated duration of 10 000 s. During the simulation, the suction compartment was kept fixed to investigate the effect of the sole storage compartment. All the other boundaries were set as free, with axial symmetry imposed on the central axis of the cup. Furthermore, the influence of air trapped inside the device was assumed to be negligible. Full compression conditions were achieved when the distance between the inner walls of the storage compartment reached 0.1 ± 0.05 mm. Contact conditions were added at relevant boundaries and penalty factors were adjusted to account for the compression forces (Table S6, Supporting Information).

### Device's Fabrication Via Cast‐Molding

To fabricate the devices, the PDMS base and curing agent were mixed at a 10 to 1 ratio (w/w) for ShA 28 and ShA 50 silicone and at a 1 to 1 ratio (w/w) for ShA 33 silicone. After a homogeneous mixing, the PDMS pre‐polymer was degassed under vacuum for 10 min. Subsequently, the mixture was poured onto the molds and subjected to a further 5–10 min of vacuum degassing (Figure S3B, Supporting Information). Following this, the stamps were inserted and the resulting merged molding plates were secured within a custom‐made, 3D‐printed, PLA holder (as previously described) using four bolts and nuts to ensure uniform pressure application during the PDMS hardening (Figure S3C, Supporting Information). Specifically, PDMS was allowed to cure overnight at room temperature and the process was always carried out with two molds simultaneously. Afterward, the stamps were removed (Figure S3D, Supporting Information) and the excess material was trimmed away. The two similar and clean molds were coated with a thin layer of freshly prepared and degassed PDMS pre‐polymer and merged (Figure S3E, Supporting Information). Similar to the previous step, the combined molds were placed into the holder, sealed, and allowed to harden overnight. Finally, the molds were opened, the devices were unmolded and any excess material was removed to obtain the final device (Figure S3F, Supporting Information).

### Tissue Preparation for the Ex Vivo Adhesion Test

Freshly extracted porcine cheek skin, from animals aged 5 to 6 months with weights ranging from 100 to 120 kg, was obtained from the local slaughterhouse (Schlachtbetrieb Zürich AG). The skin was immediately immersed in DMEM/F12 medium supplemented with 1% (v/v) Antibiotic‐Antimycotic 100X and kept on ice within 15 min of extraction. No later than 2 h after extraction, the tissue was processed by removing excess fat and muscle tissue to trim it to a thickness of ≈1.5 cm. The tissue was cut into pieces without causing damage to the skin and affixed to a 4 × 4 cm 3D‐printed platform (Figure S24A, Supporting Information). This platform was designed in SolidWorks and DLP printed (Asiga Max 27 UV, Asiga) with a commercial resin (Asiga Plasclear, Asiga). Prior to starting the adhesion test, the skin tissue was gently shaved to ensure complete removal of hair.

### Adhesion Test

The adhesive properties of the blood sampling devices were assessed by measuring the pull‐off force using a texture analyzer (TA.XT plus Texture Analyser, 50 kg load cell, Stable Micro Systems). To perform the analysis, a needle was inserted into the device's knob to facilitate the attachment of a cotton thread connecting the device to the pulling clamp of the instrument. Freshly extracted porcine skin, prepared as previously described , was used as the 0° platform for the adhesion test and securely fastened to the lower pulling clamp of the instrument (Figure S24B, Supporting Information). To ensure proper sealing of the device, two drops of a 2% (w/v) hydroxyethyl‐cellulose solution in distilled water were applied to the skin's surface. Subsequently, the instrument was pulled until the device's detachment at a rate of 5 mm s^−1^. Pulling force and distance were recorded using the Exponent software (Stable Micro Systems).

### Negative Pressure Measurements

To determine the negative pressure generated by device compression, an in‐house setup was fabricated (Figure S25A,B, Supporting Information). The setup comprised a 3D‐printed platform designed using SolidWorks, which was then fabricated using an LCD printer (Jenny Light 1+) and a Value Line UV/DLP Tough (ABS‐like) clear resin. After printing, the platform was washed twice with isopropyl alcohol in a sonicator bath at 40 °C for 30 min and then UV‐cured for 2 h. A 4‐mm thick silicone layer (13 ShA) was attached to the top of the platform, whilst an Adafruit MPRLS ported pressure sensor breakout (0 to 25 PSI) (Adafruit Industries) connected to a Raspberry Pi microcontroller (Raspberry Pi 4 Computer, Model B 4GB RAM, Raspberry Pi Foundation) was affixed to the bottom of the setup. A small hole was drilled at the center of the setup to enable a tubing connection between the sensor and the silicone layer. For recording negative pressure, the cast‐molded devices were manually compressed in the air and then placed on top of the silicone layer. Pressure changes were recorded throughout the process. Minor vacuum leakage occurred during the experiment due to the limitations of the measuring platform.

### Force‐displacement Measurements

To evaluate device displacement during compression, a TA.3DXTplus texture analyzer (Stable Micro Systems) equipped with a 50 N load cell was used. For the compression surface, a custom‐designed 3D‐printed platform, created using SolidWorks (Dassault Systemes SE) and manufactured with an LCD printer (Jenny Light 1+) and Value Line UV/DLP Tough (ABS‐like) clear resin, was utilized (Figure S26, Supporting Information). After the cleaning and curing process, a 2‐mm diameter hole was drilled in the platform. This hole was strategically placed to facilitate the release of air from the device during compression analysis. The instrument was then calibrated and the device was positioned on the platform's surface. The experiment was performed at a controlled compression speed of 1 mm s^−1^. During the experiment, force/displacement curves were recorded, and the device's displacement values at a compression force of 30 N (equivalent to manual compression force) were extrapolated. The measurements were performed in triplicate.

### In Vitro Fluid Extraction Experiments

The liquid extraction performance of the optimized cast‐molded device was evaluated using three different fluids: DW, BMF, and porcine whole blood treated with sodium oxalate as an anticoagulant, freshly obtained from a local slaughterhouse (Schlachtbetrieb Zürich AG). The BMF was prepared by mixing 40% w/v glycerol and 5% w/v KI in distilled water, achieving a viscosity range of 3.5 to 5.5 mPa·s. To perform the experiment, a custom 3D‐printed cell was designed and LCD 3D‐printed (Jenny Light 1+) with a Value Line UV/DLP Tough (ABS‐like) clear resin. The cell was then provided with a plastic petri dish onto which a 4‐mm thick layer of silicone (13 ShA) was glued. This soft silicone layer was used as a human skin surrogate, ensuring proper device adhesion. Subsequently, a 29‐G hole (*d =* 0.184 mm) was drilled in the silicone‐petri dish and the cell was entirely filled with the extraction fluid and its lateral arm was sealed with parafilm. A schematic representation of the complete setup for the in vitro fluid extraction experiment is shown in Figure S10A (Supporting Information). Simultaneously, the optimized device was equipped with adhesive tape (Double Coated Silicone/Acrylic tape, MKB tape solutions) to ensure proper adhesion to the silicone layer of the 3D‐printed cell. Finally, the device was manually compressed on the setup surface. After a 10 min application, the device was detached and the extracted volume was measured using a 100‐µL pipette. To better replicate the complexity of in vivo blood sampling, the setup for the in vitro fluid extraction experiment was slightly modified. Specifically, a crosslinked and highly porous gelatin matrix was introduced into the 3D‐printed cell and 20 mL of porcine whole blood, treated with an anticoagulant, was added. The experiment was then conducted as previously described. The gelatin matrix was prepared by dissolving 4 g of gelatin in 100 mL of distilled water (final gelatin concentration 4% w/v) at 50 °C under magnetic stirring. Subsequently, the gelatin solution was chemically crosslinked by adding 0.5% v/v of a 25% glutaraldehyde aqueous solution and the resulting crosslinked hydrogel was frozen at −20 °C overnight to facilitate the formation of a cryogel with a highly porous structure.

### Tissue Preparation For the Ex vivo Nimble Experiment and Device Application

Freshly extracted porcine cheek skin, obtained from animals aged 5 to 6 months with weights ranging from 100 to 120 kg, was obtained from the local slaughterhouse (Schlachtbetrieb Zürich AG, Zürich, Switzerland). The skin was immediately immersed in DMEM/F12 medium supplemented with 1% (v/v) Antibiotic‐Antimycotic 100X and kept on ice within 15 min of extraction. Within one hour of extraction, the tissue was placed on ice and gently shaved to completely remove the hair. Subsequently, it was rinsed and pat‐dried before conducting the experiments. For the Nimble experiments, multiple test sites were selected on a large tissue sample devoid of visible scars or visual damage. For ex vivo device application, sites measuring ≈30 × 30 mm with similar properties were cut from the tissue, ensuring they were free from any prior damage.

### Ex vivo Nimble Experiments

To assess skin properties, an ultra‐light, suction‐based skin stiffness evaluation instrument proposed by Müller et al. was used.^[^ ^56^ ^]^ Briefly, this apparatus consisted of a probe to which one vertical and one horizontal hollow metal pins were glued. These pins allowed the connection of the probe to a pressure sensor via tubing. Specifically, the vertical pin was directly linked to the pump responsible for the constant increase of the applied pressure, whilst the horizontal one served as a reference. Self‐made probes, with a specific height of 2, 2.5, 3, 3.5, and 4 mm, and a fixed diameter (equal to that of the optimized device) were designed in SolidWorks (Dassault Systemes SE) and LCD 3D‐printed (Jenny Light 1+) using a Value Line UV/DLP Tough (ABS‐like) clear resin. After printing, the metal pins were glued to these customized probes. Finally, the cups were gently applied on the freshly extracted porcine skin (prepared as previously described ) and the measurement was started. A pressure ramp of 0.3 kPa/s was constantly applied by the instrument. The measurement automatically stopped once a pressure difference of 1 kPa between the measurement and the reference pin was recorded. The measurements were performed in two independent experiments (two cheeks) and each experiment was carried out with nine replicates (*n* = 18).

### Ex vivo Device Application to Determine the Height of the Stretched Skin

The final device was manually compressed on the porcine skin. After 5 min application, the porcine skin, together with the compressed device was snap‐frozen in a nitrogen‐cooled isopentane (2‐methylbutane, Sigma Aldrich) bath for a few minutes. After freezing, the device was removed and the skin sample was embedded in an optical cutting temperature compound (OCT, Leica microsystems). The embedded tissue was frozen at −20 °C for 24 h and then stored at −80 °C. Transversal sections of 20 µm thickness were obtained by cryostat sectioning (CryoStar NX50, Thermo Fisher Scientific) at −18 °C and placed on microscope slides (Thermo Scientific), which were stored at −80 °C until further processing.

### Ex vivo Histological Evaluation of the Sucked Porcine Skin After Device Application

Microscope slides were thawed for 30 min at RT. Afterward, they were fixed with a 4% w/v methanol‐free PFA solution in PBS for 10 min, followed by a washing step in PBS for 3 min. To evaluate tissue morphology, fixed sections were stained with H&E. Briefly, they were stained with Mayer's hematoxylin (Medite AG) for 3 min, followed by a 1 min wash under a gentle stream of tap water and a 20 s immersion in HCl‐EtOH mixture (700 mL absolute EtOH + 299 mL distilled water + 1 mL 37% HCl) to blue the stain. The slides were then rinsed under a gentle stream of tap water for 1 min before proceeding to the Eosin Y (Chroma‐Gesellschaft) staining step. Following this, the slides were dehydrated and cleared by subsequent immersions in solutions with an increased EtOH concentration (2 × 95% EtOH, 2 × 100% EtOH) and xylene (Merck KGaA) (2x), respectively. Finally, they were mounted with ProLongTM Diamond Antifade Mountant (Invitrogen) and covered with a coverslip. Cross‐sectional images were recorded using the Leica DMI 6000 B microscope with adaptive focus control (annual maintenance by Leica support service, Leica Microsystems) at a magnification of ×20 (HC PL FLUOTA R L 20x, with correction collar (CORR), numerical aperture: 0.4, dry, Leica Microsystems) using the LAS X software (Leica Microsystems) in the tile scan mode (manual and automatic focusing; range, 60 µm) and were merged afterward. The light was emitted by an E L6000 mercury metal halide bulb (Leica Microsystems). Images were detected by a monochrome D FC365FX digital camera (12 bits, 1 × 1 BIN, Leica microsystems).

### MN Array Fabrication

For the fabrication of the MN array, circular basins with a diameter of 8.4 mm and an overall height of 3 mm were designed in SolidWorks (Dassault Systemes SE) and then DLP printed (Asiga Max 27 UV, Asiga) with a commercial resin (Asiga Plasclear, Asiga). For the circular MN arrays, basins featured a single circular channel with a diameter of 3.5 mm or two concentric circular channels with an outer and an inner diameter of 3.5 mm and 2.1 mm, respectively. These basins were fabricated to accommodate 4 blades (20 MNs in total) or 6 blades (30 MNs in total), respectively. Instead, for the rectangular MN arrays, basins were equipped with 4 parallel channels with an inter‐channel distance of 1.2 mm. The channel width was set to 0.40 mm for both the basin's geometries. Thereafter, stainless steel Bl‐2 and Bl‐3 were glued to the basin's channels with a UV Glue (Permabond UV and Visible Light Cure Adhesive, Permabond Engineering Adhesives Ltd) after 10 min of UV curing. To craft circular MN arrays, the blades were first manually bent around a stainless‐steel cylinder ( *d* = 2 mm) before being glued to the basin's channels.

### Penetration Force of MN Blades

The mechanical properties of Bl‐2 and Bl‐3 were assessed on a single MN. Specifically, four out of five MNs were removed from each blade, retaining only the central MN. The MN was then embedded in the central channel of the 3D‐printed basin (as previously described) and subsequently dipped in a dye solution. Afterward, the MN penetration force was measured with a texture analyzer (TA.XT plus Texture Analyser, 50 kg load cell, Stable Micro Systems). Indeed, the basin was attached with a double‐sided tape to the cylindrical compression probe of the instrument with the MN facing down, whilst a freshly extracted porcine skin (prepared as previously described) was used as a platform for the penetration test and fixed to the lower clamp of the instrument. To reduce the intrinsic variability of different skin samples the force‐displacement test was performed on two adjacent pieces of freshly extracted porcine skin of the same animal. The penetration test was performed at a speed of 0.5 mm s^−1^. Force‐displacement measurements were acquired from the point at which the sensor first touched the MN tips until reaching a fixed displacement value of 1.5 mm. Notably, no buckling of the MNs was observed during this period. Finally, at the end of the test, cross‐sectional incisions were made in the porcine skin to confirm the penetration of the MNs. The penetration force was determined as the peak value on the force‐displacement curve, occurring before a sudden decrease.

### PLA Plate Fabrication

A PLA plate was developed to facilitate full compression of the device to ensure complete exposure of the MN array during the device's application. Specifically, the PLA plate was designed in SolidWorks (Dassault Systemes SE) and then 3D‐printed via Fused Deposition Modeling (FDM) (Pro 2, Raise 3D Technologies, Inc.) using PLA (SPECTRUM Filament PLA tough natural, 1.75 mm). The dimensions of the plate included a y‐ and an x‐height of 40.6 mm and 48.8 mm, respectively. Finally, it featured a circular hole with a diameter of 20.5 mm, designed to accommodate the bottom rim of the cast‐molded device.

### Device Combination with MN Patch for Ex Vivo Skin Puncturing Experiments

MN arrays composed of 4 Bl‐2 and Bl‐3 (prepared as previously described) were dipped in a dye solution (Tissue Marking Dye, blue, Diapath) and allowed to dry overnight. Thereafter, the patches were attached to the inner dome of the optimized device using fresh silicone (Sil‐Poxy/0 Silicone Adhesive). Finally, the bottom rim of the silicone device was treated with a small amount of Permabond POP primer (polyolefin primer, Permabond Engineering Adhesives Ltd) to allow the attachment of the 3D‐printed PLA plate with cyanoacrylate glue (Permabond 2050 Flexible superglue, Permabond Engineering Adhesives Ltd). The assembled devices were stored under ambient conditions until further use.

### Ex vivo Assessment of MN Penetration Depth

To assess the ability of the MNs to penetrate the skin, the assembled device comprising the MN patch and the PLA plate was manually compressed on the porcine cadaver ear skin for 10 s. Subsequently, the device was removed, any excess dye resulting from MN insertion was wiped with distilled water and the skin was imaged with an optical microscope (Leica MZ6 equipped with Flexcam C3) to identify residual holes. Afterward, to determine the penetration depth of the MNs, the punctured skin was frozen in an optical cutting temperature compound (OCT, Leica microsystems) and then sectioned in 20 µm slices using a cryostat (CryoStar NX50, Thermo Fisher Scientific) at −18 °C. Slices were then placed on microscope slides (Thermo Scientific), mounted with ProLongTM Diamond Antifade Mountant (Invitrogen), and covered with a coverslip. Finally, cross‐sectional images were taken using the Leica DMI 6000 B microscope.

### Ex vivo Histological Evaluation of the Porcine Ear Skin After MN Puncturing

Microscope slides were thawed for 30 min at RT. Subsequently, they were fixed using a 4% PFA solution and subjected to the H&E staining procedure as described above. Histological examination was conducted by Leica DMI 6000 B microscope under bright‐field illumination.

### PLA Lid and Adapter Fabrication

A PLA lid and adapter were designed in SolidWorks (Dassault Systemes SE) and then 3D‐printed via Fused Deposition Modeling (FDM) (Pro 2, Raise 3D Technologies, Inc.) using PLA (SPECTRUM Filament PLA tough natural, 1.75 mm). 3D cross‐section designs are reported (Figure S27, Supporting Information). A minute capillary was inserted into the tip of the PLA adapter.

### Blood Storage Stability Study

Five hundred µL of human whole blood were loaded into the storage compartment of the PI. Subsequently, the tailored 3D‐printed lid was inserted to ensure proper sealing. The PI, with the lid facing upward, was then placed in a sealed bag and stored at RT for a 3‐day period. At predefined intervals (0, 1, 2, and 3 days), the sealed bag was opened, the lid removed and both the blood volume within the storage compartment and its pH were measured. Human whole blood treated with K2‐EDTA (pooled, mixed gender) was shipped and stored at −80 °C. Blood samples were collected under ISO, FDA, USDA, and EPA certificates.

### Device Combination With a Commercial Malaria Paper Test

Five hundred µL of whole human blood were spiked with 2 µL of recombinant PfHRP2 (2.25 mg/mL) and loaded in the storage compartment of the PI. Thereafter, the PI was coupled with the tailor‐made adapter and one drop of blood was drop‐cast on the malaria test strip area after device compression. Furthermore, three drops of the buffer solution (provided with the commercial kit) were added, allowing the blood to flow through the test strip. After a few minutes, the test result was displayed.

### Device Assembling for the in Vivo Study

Two prototypes featuring 20 or 30 MNs (PI‐20 and PI‐30) were selected for the in vivo study and assembled as previously described. Subsequently, 150 µL of a 1% w/V K_2_‐EDTA solution in a 1:1 volume ratio mixture of EtOH and distilled water were introduced into the device's storage compartment and allowed to dry overnight at RT. The resulting devices were sterilized via autoclaving (121 °C, 20 min). Thereafter, an electrocardiogram adhesive tape (ECG), previously sterilized through UV light exposure for 30 min, was affixed to the underside of the device using fresh silicone adhesive (Sil‐Poxy/0 Silicone Adhesive). To minimize the risk of contamination, this step was performed within a laminar flow cabinet. Finally, the devices were transferred into a sterile plastic container before being shipped for the in vivo study.

### In vivo Study on Piglets

The in vivo protocol was ethically approved by the Centre National de Biologie Expérimentale (CNBE) and the Institutional Animal Protection Committee (CIPA) under the number 2212‐03 (modification 2023‐02). In vivo experiments were carried out at the INRS (CNBE, Canada). Two piglets, aged between 3 to 5 weeks and weighing ≈6–10 kg, were used in the study. Before the application of the device, the collection site was gently shaved using a razor to ensure a hair‐free surface. Subsequently, the area was cleaned with an alcohol pad to maintain sterility and finally, it was gently warmed with a heat pack for 1 min to increase the blood flow. The devices were then weighed (W_D_t_0_) and applied to the lower flank on the side of the piglet. A proper device application involved firmly pressing down the device's knob with both hands until the PLA plate was reached. This allowed complete exposure of the MN arrays (Figure S18, Supporting Information). The device was affixed to the piglet's skin while in its fully compressed configuration. To ensure an airtight seal, the ECG tape was carefully sealed to the porcine skin before releasing the device. After a 10 min application, the device was removed and its weight was recorded (W_D_t_1_). Finally, the collected blood was transferred into an Eppendorf tube and the device was once again weighed (W_D_t_2_). The amount of collected blood was determined according to Equation 1:

(1) $$\mathrm{Collected}\mathrm{blood}\left(\mu L\right)=\left[\left(W_{D}t_{2}-W_{D}t_{1}\right)/\rho \right]\times 1000$$

where ρ is the blood density at 25 °C.

To assess any residual blood inside the storage compartment, each device was cut in half. Images were taken before device application and at different time points after device removal from 0 to 3 days after the start of the experiment to monitor the evolving conditions of the piglet's skin.

### Statistical Analysis

All results were presented as mean ± SD. P‐values were calculated using an unpaired t‐test with a two‐tailed distribution and unequal variance. *p < 0.05, ^**^ *p <* 0.01, ^***^ *p <* 0.001, ^****^ *p <* 0.0001. For comparison of more than two groups, one‐way ANOVA with post‐hoc Tukey HSD Test was used. For comparison of more than two groups with two variables involved, two‐way ANOVA with post‐hoc Tukey HSD Test was used.

## Conflict of Interest

The authors declare no conflict of interest.

## Supporting information

Supporting Information

Supplemental Video 1

Supplemental Video 2

## Data Availability

The data that support the findings of this study are available in the supplementary material of this article.
