# Supplementary material for: A Bioinspired and Cost‐Effective Device for Minimally Invasive Blood Sampling
Source: Adv Sci (Weinh). 2024 Mar 7;11(18):2308809. doi: 10.1002/advs.202308809 (PMC11095219; doi:10.1002/advs.202308809)
Supplement: Supplementary file 1 — Supporting Information [file ADVS-11-2308809-s001.pdf]

ADVANCED SCIENCE
Open Access

Supporting Information

for *Adv. Sci.*, DOI 10.1002/advs.202308809

A Bioinspired and Cost-Effective Device for Minimally Invasive Blood Sampling

*Nicole Zoratto, David Klein-Cerrejon, Daniel Gao, Tino Inchiparambil, David Sachs, Zhi Luo and Jean-Christophe Leroux**

A bioinspired and cost-effective device for minimally invasive blood sampling

Nicole Zoratto[1], *David Klein-Cerrejon*[1], *Daniel Gao*[1], *Tino Inchiparambil*[1], *David Sachs*[2], *Zhi Luo*[3], *Jean-Christophe Leroux*[1*]

[1]Institute of Pharmaceutical Sciences, Department of Chemistry and Applied Biosciences, ETH Zurich, Zürich, 8093 Switzerland.

[2]Institute for Mechanical Systems, Department of Mechanical and Process Engineering, ETH Zurich, Zürich, 8093 Switzerland.

[3]Department of Biomedical Engineering, Southern University of Science and Technology, Shenzhen 518055, Guangdong, P.R. China.

*E-mail: jleroux@ethz.ch

This file includes:

Figures. S1 to S24

Tables S1 and S5

Other Supplementary Material for this manuscript includes the following:

Movies S1 and S2

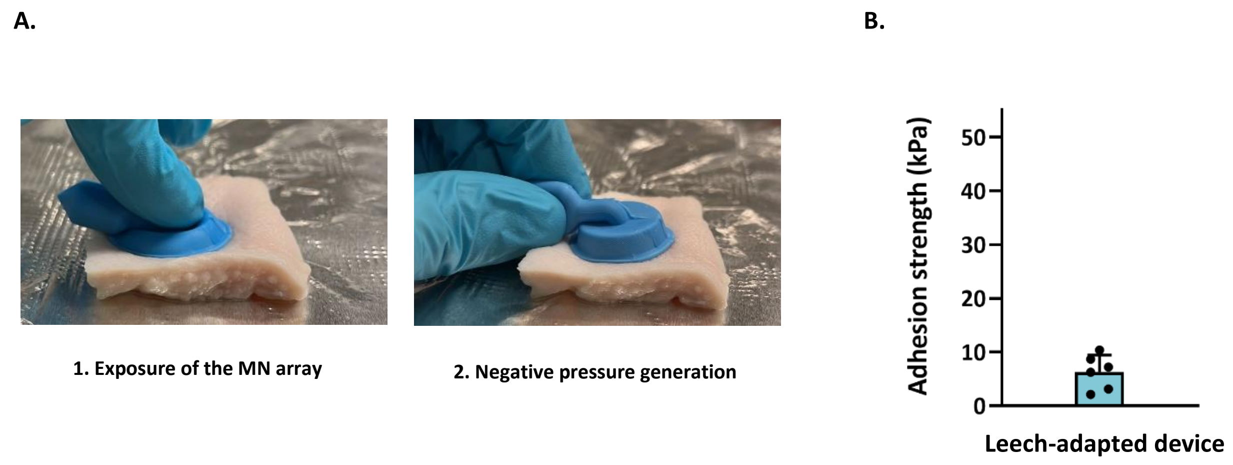

Figure S1. (**A.**) Direct leech-adapted design featuring a suction cup and a fluid storage compartment connected by a 90° channel. The device requires a two-step application for collecting capillary blood. First, a compression of the suction cup exposes the MN array, enabling capillary puncture (left). Then, a second compression of the storage compartment creates negative pressure to enhance blood withdrawal (right). (**B.**) Adhesion strength of the leech-

adapted device. The study involved manually compressing the device onto fresh extracted porcine skin and measuring adhesion strength during device pull-off at a 0° angle. Data are expressed as mean + SD (*n* = 6).

Table S1. List of the devices investigated in the design optimization study. For each device, the 2D sketch illustrating the proportional dimensions, 3D cross-section view, hardness (ShA) of the silicone used during the fabrication process and volumes are reported. V_T , V_{ST}, V_{SC} represent the total inner volume, the volume of the storage compartment and the volume of the suction cup, respectively. $r_{ST/SC}$ is the ratio between the storage compartment and suction cup volumes.

Device	Sketch	3D cross-section	Silicone type (ShA)	Volume (cm³)
1	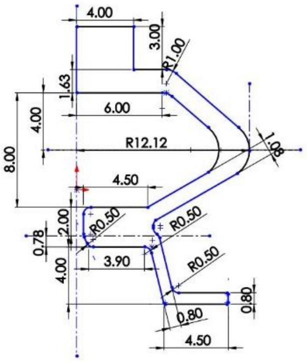 	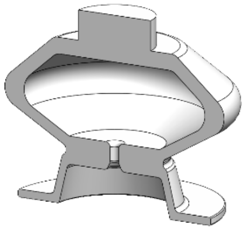	28	V_T = 2.21<br>V_{ST} = 1.81<br>V_{SC} = 0.4<br><br>$r_{ST/SC}$ = 4.5
2	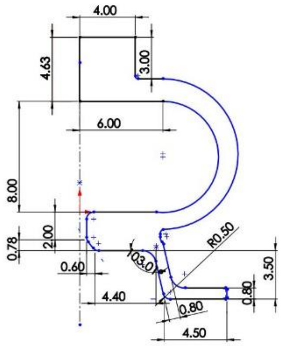 	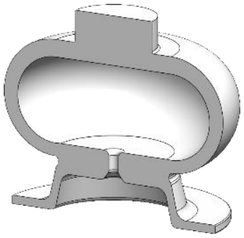	28	V_T = 2.49<br>V_{ST} = 2.13<br>V_{SC} = 0.36<br><br>$r_{ST/SC}$ = 5.9
3	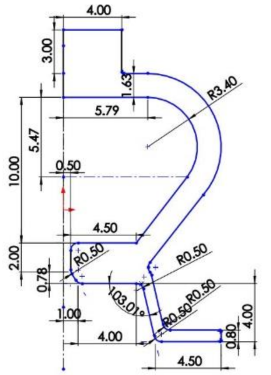 	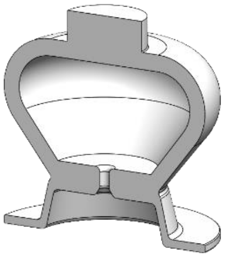	28, 33, 50	V_T = 2.39 V_{ST} = 1.95<br>V_{SC} = 0.44<br><br>$r_{ST/SC}$ = 4.4

4	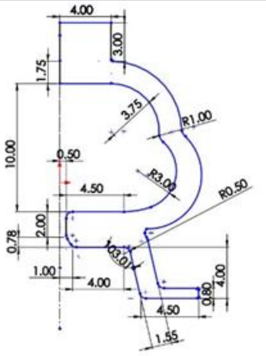	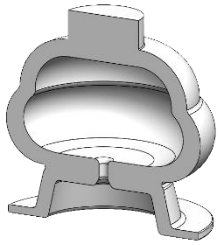	28, 33, 50	$V_T = 2.41$<br>$V_{ST} = 1.97$<br>$V_{SC} = 0.44$<br><br>$r_{ST/SC} = 4.5$
5	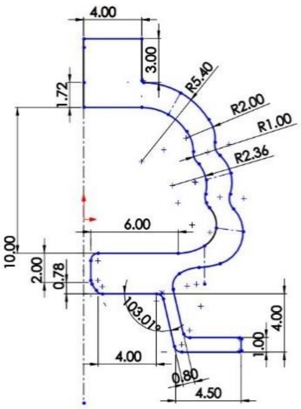	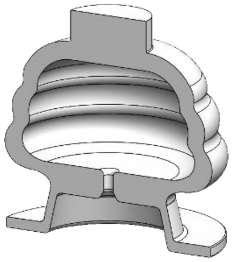	28	$V_T = 2.43$<br>$V_{ST} = 2.0$<br>$V_{SC} = 0.43$<br><br>$r_{ST/SC} = 4.7$
6	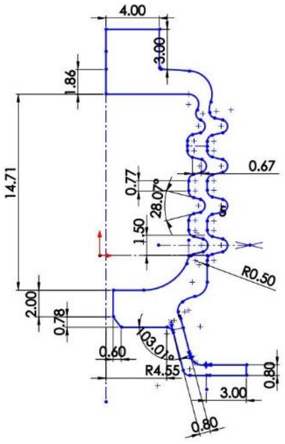	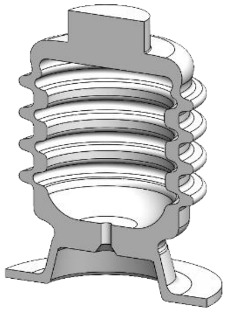	28	$V_T = 2.33$<br>$V_{ST} = 1.99$<br>$V_{SC} = 0.34$<br><br>$r_{ST/SC} = 5.9$

7	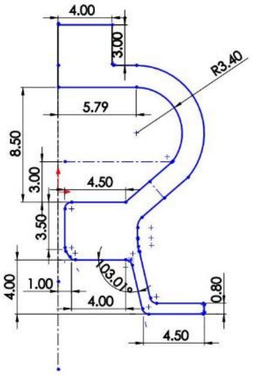	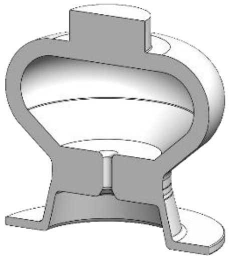	50	$V_T = 2.17$<br>$V_{ST} = 1.74$<br>$V_{SC} = 0.43$<br><br>$r_{ST/SC} = 4.0$
8	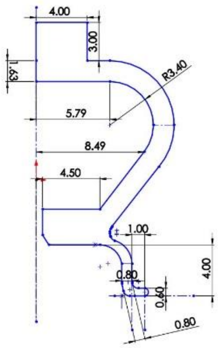	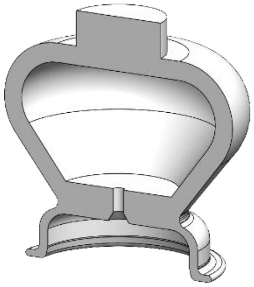	50	$V_T = 2.50$<br>$V_{ST} = 1.96$<br>$V_{SC} = 0.54$<br><br>$r_{ST/SC} = 3.6$
9	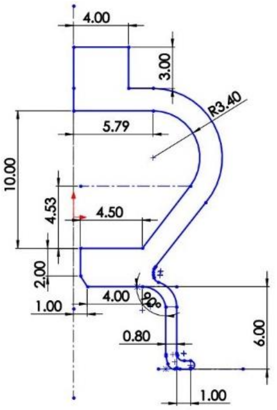	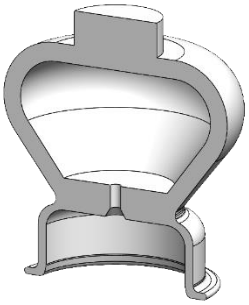	50	$V_T = 2.78$<br>$V_{ST} = 1.95$<br>$V_{SC} = 0.83$<br><br>$r_{ST/SC} = 2.3$

10	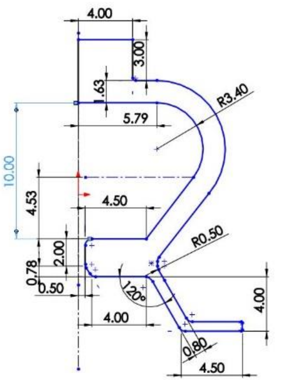	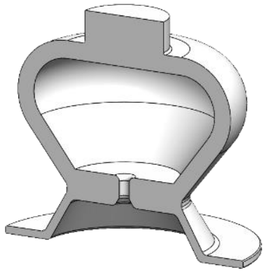	50	V_T = 2.48<br>V_{ST} = 1.41<br>V_{SC} = 1.07<br><br>$r_{ST/SC}$ = 1.3
11	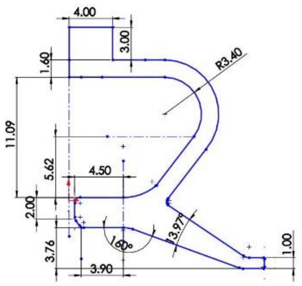	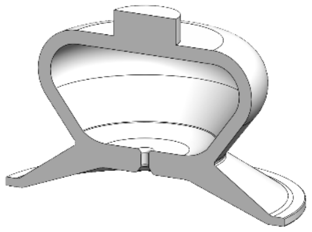	50	V_T = 5.32<br>V_{ST} = 3.9<br>V_{SC} = 1.42<br><br>$r_{ST/SC}$ = 2.7
12	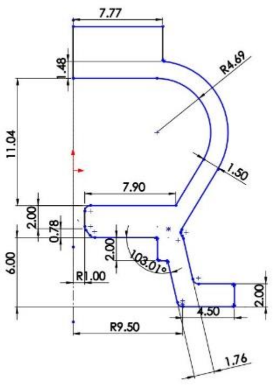	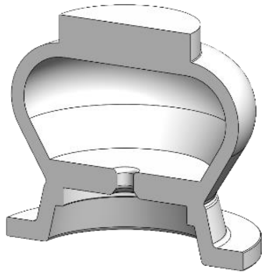	50	V_T = 5.4<br>V_{ST} = 4.09<br>V_{SC} = 1.31<br><br>$r_{ST/SC}$ = 3.1
13	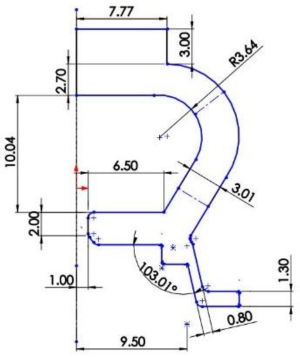	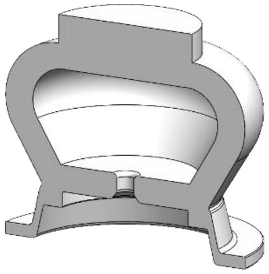	50	V_T = 4.36<br>V_{ST} = 2.9<br>V_{SC} = 1.46<br><br>$r_{ST/SC}$ = 2

14	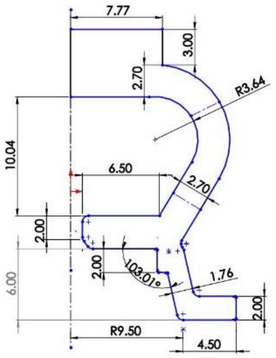	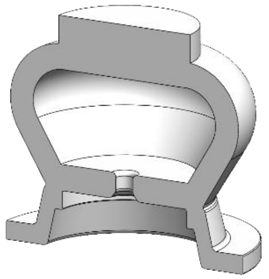	50	V_T = 4.22<br>V_{ST} = 2.94<br>V_{SC}= 1.28<br><br>$r_{ST/SC}$ = 2.3

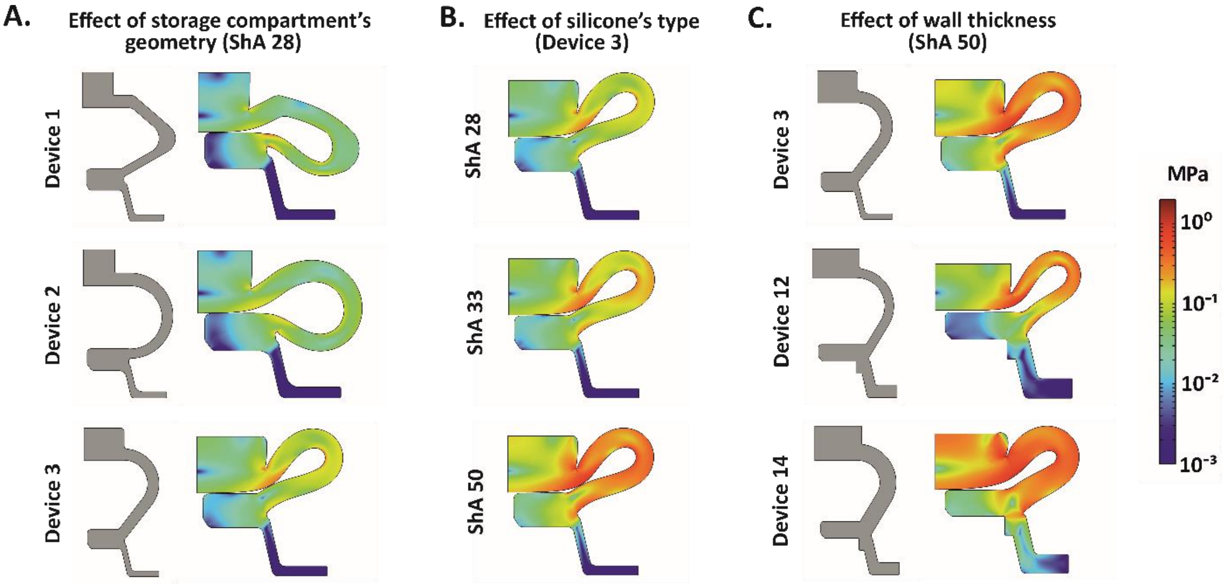

Figure S2. Effect of (**A.**) storage chamber's geometry, (**B.**) silicone ShA hardness and (**C.**) wall thickness on the Von-Mises stress distribution profiles at compression for different microsampling devices, as calculated through FEM simulation. Maximum compression was defined as occurring when the distance between the inner walls of the storage compartment reached 0.1 mm.

Table S2: Average strain density energies and residual air volumes within the later pockets of the devices, as calculated through FEM simulation.

	**Average strain density energy (J/mm^3)**	**Residual air volume (cm^3)**
**Device 1 (ShA 28)**	1.87 x 10^{-6}	0.56
**Device 2 (ShA 28)**	2.02 x 10^{-6}	0.83
**Device 3 (ShA 28)**	6.72 x 10^{-6}	0.32
**Device 3 (ShA 33)**	9.78 x 10^{-6}	0.32
**Device 3 (ShA 50)**	20.06 x 10^{-6}	0.32
**Device 12 (ShA 50)**	10.70 x 10^{-6}	0.77
**Device 14 (ShA 50)**	28.60 x 10^{-6}	0.45

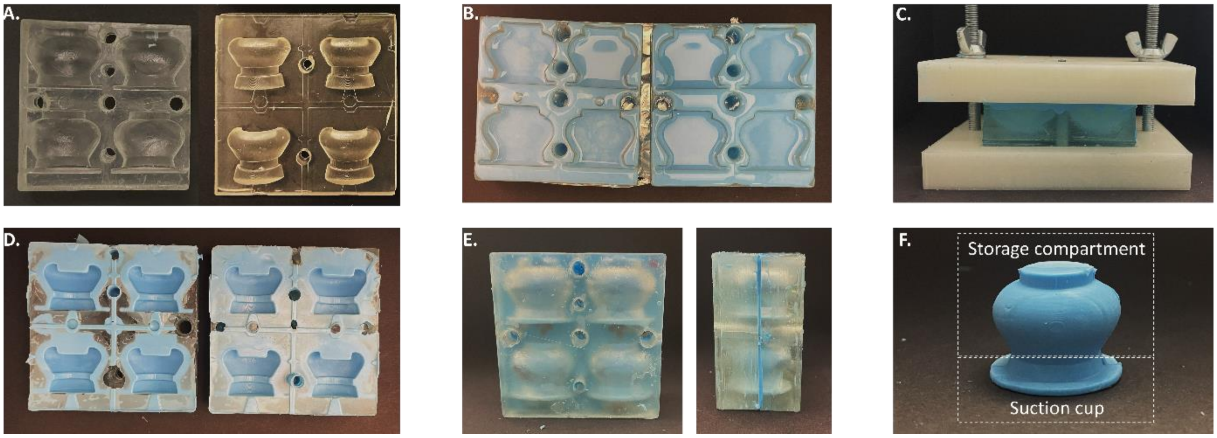

Figure S3. (**A.**) LCD 3D-printed negative casting molds with 2 x 2 patches. Workflow for the microsampling device fabrication: (**B.**) Casting of PDMS inside the molds followed by the insertion of the stamps for the inner structure. (**C.**) Merged molding plates tightened together in a custom-made holder with four bolts and nuts to uniformly apply pressure overnight (image taken from the side view). (**D.**) Two molds with the cross-linked half structure of the device and the excess material from overflow channels after the removal of the stamps. (**E.**) Two identical molds, after the removal of the excess material and the subsequent coating with a freshly prepared PDMS, merged to form the full blood microsampling device. The merged molds were tightened together in a custom-made holder with four bolts and nuts to uniformly apply pressure overnight. The image on the right shows the side view of the two merged and solidified half structures. (**F.**) Final blood microsampling device after the demolding and the removal of residual PDMS. The terms “suction cup” and “storage compartment” were introduced to differentiate the functions of these two integrated chambers.

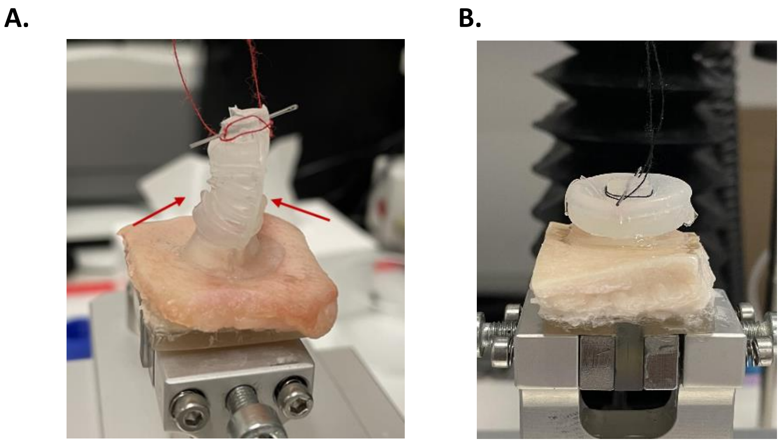

Figure S4. Photograph of devices 6 and 3 during *ex-vivo* adhesion test. The original idea of the accordion-like structure was to facilitate complete air removal during compression. Yet, due to design constraints, it did not allow vertical compression in contrast to the other tested devices (**A.**). As a comparison, a photograph of device 3 in the same ex-vivo adhesion test is reported (**B.**).

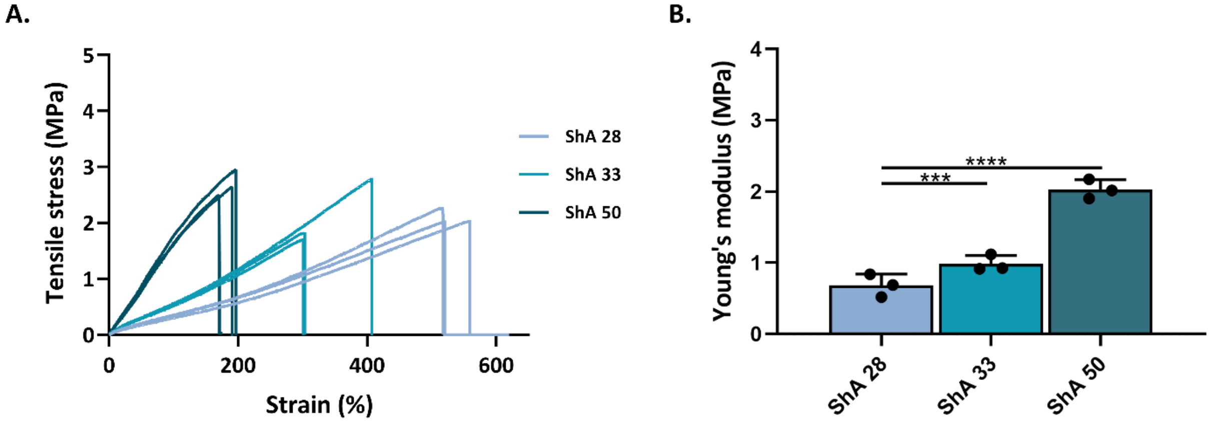

Figure S5. (**A.**) True stress-strain curves and (**B.**) average Young's moduli obtained in a tensile test with cast-molded dog-bone-shaped specimens composed of ShA 28 (light blue), ShA 33 (turquoise) and ShA 50 (dark teal) silicone. The tensile properties are expressed as mean + SD (*n* = 3). Statistical significance was calculated by one-way analysis of variance (ANOVA) with Tukey's comparison test with ***P < 0.001 and ****P < 0.0001.

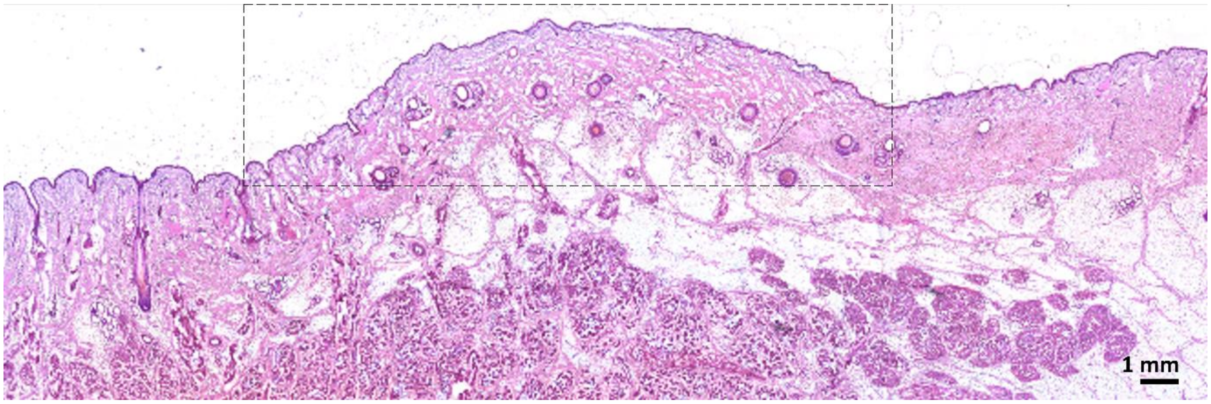

Figure S6. *Ex-vivo* porcine skin stained with H&E after 5-min application of the blood sampling device. The highlighted bump area, delineated by a dashed rectangle, indicates the tissue drawn into the device. Scale bar: 1 mm.

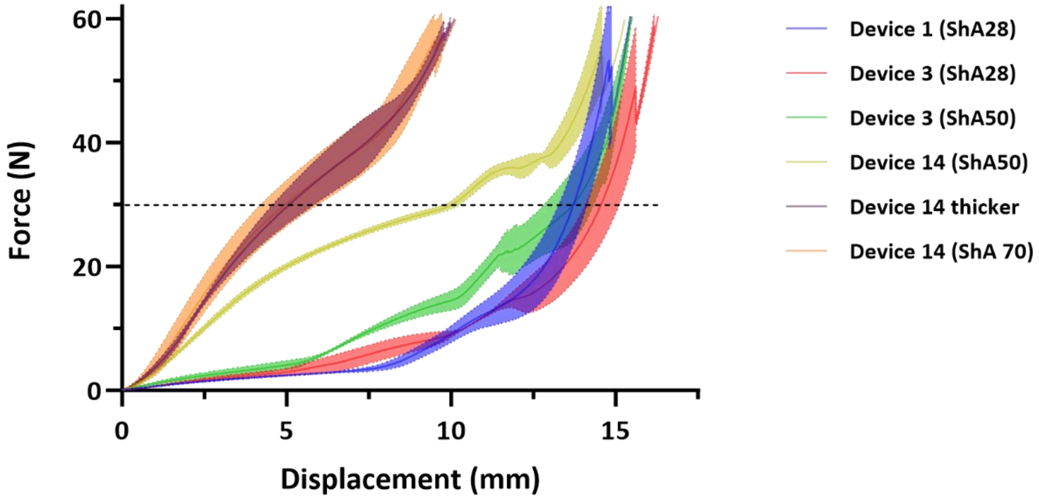

Figure S7. Compression profiles for various device designs. Experimental data are expressed as mean ± SD (n = 3). Increasing the device's wall thickness or the silicone ShA hardness resulted in a reduced device's displacement. The dashed line represents a compression force of 30 N, chosen to simulate manual compression force.

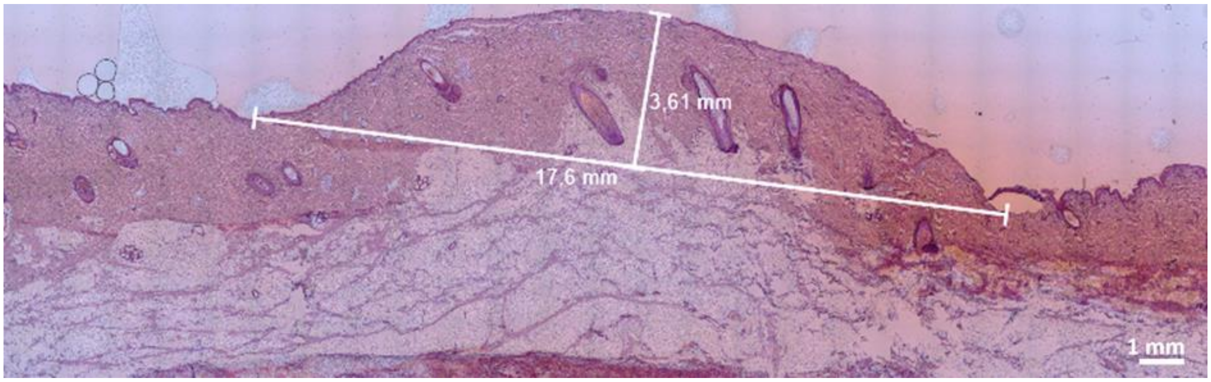

Figure S8. Ex-vivo porcine skin stained with H&E after 5-min application of device 14. A skin deformation of ca 3.6 mm was observed. This stretching of the skin, coupled with the 2 mm length of the MNs and the inclusion of a 1 mm minimum-height basin for MN embedment, yielded a total of 6.6 mm. Therefore, positioning the MN patch directly on the septum (situated exactly 6 mm from the device's bottom) may result in an incomplete retraction from the skin upon the device's elastic recovery.

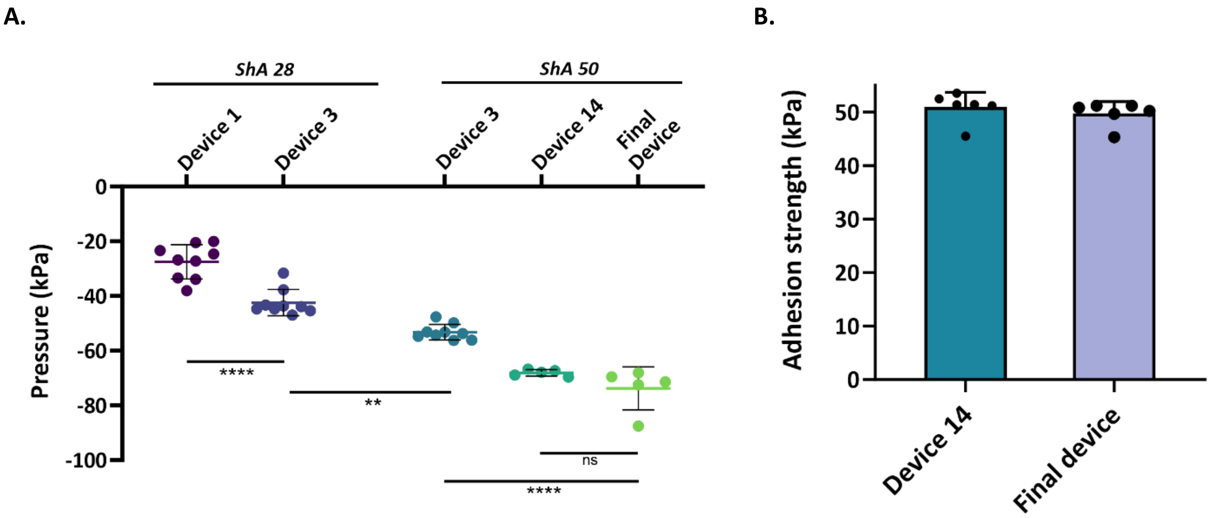

Figure S9. (**A.**) Negative pressure generated by the compression of the final device as compared to that generated by previous prototypes. The best negative pressure value was recorded for the final, optimized device (-73.8 ± 7.9 kPa). Experimental data are expressed as mean + SD (n = 5-9). Statistical significance was calculated by one-way analysis of variance (ANOVA) with Tukey's comparison test with **P < 0.01, ****P < 0.0001 and ns: P > 0.05. (**B.**) Adhesion strength values of the final device *vs* device 14 at compression. Experimental data are expressed as mean + SD (n = 6). Statistical significance was calculated by unpaired t-test with a two-tailed distribution and unequal variance. (ns: P > 0.05).

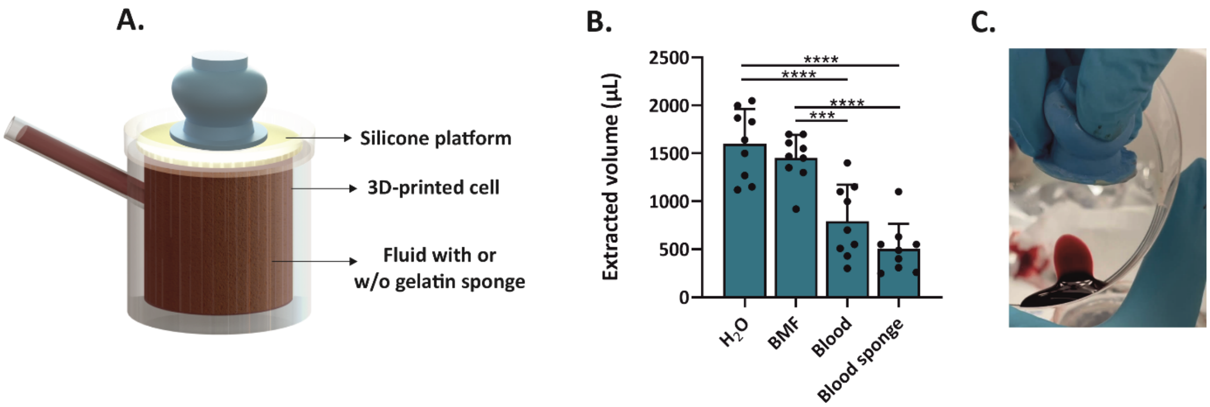

Figure S10. (**A.**) Schematic representation of the setup used for the *in-vitro* fluid extraction experiments. (**B.**) *In-vitro* extraction volumes of water, blood-mimicking fluid (BMF) and blood through the blood-extraction system with the blood sampling device after 10-min application. For blood extraction, the experiment was performed with and without the presence of a gelatin sponge into the fluid extraction setup. Data are expressed as mean + SD (n = 3-6). Statistical significance was calculated by two-way analysis of variance (ANOVA) with Tukey's comparison test with ***P < 0.001 and ****P < 0.0001. (**C.**) Blood collected after the compression of the final device following a 10-min application on the fluid extraction setup. The in-vitro extraction experiment was conducted with the presence of a gelatin sponge and anticoagulated whole porcine blood.

Table S3. Acronyms, structure and sizes of the stainless-steel blades tested for the initial MN array development. Blades Bl-1, Bl-2 and Bl-4 share identical geometries and differ exclusively in terms of thickness.

Blade name	Structure	Blade size
Bl-1	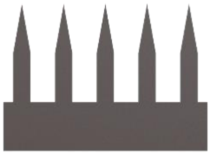	Needle length: 2.0 mm<br>Needle width: 0.35 mm<br>Needle pitch: 0.50 mm<br>Angle tip: 20°<br>Thickness: 100 µm
Bl-2	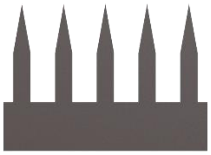	Needle length: 2.0 mm<br>Needle width: 0.35 mm<br>Needle pitch: 0.50 mm<br>Angle tip: 20°<br>Thickness: 75 µm
Bl-3	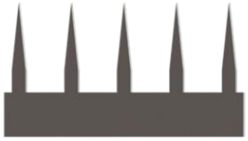	Needle length: 2.0 mm<br>Needle width: 0.35 mm<br>Needle pitch: 1.0 mm<br>Angle tip: 13.4°<br>Thickness: 75 µm
Bl-4	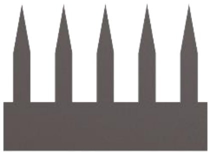	Needle length: 2.0 mm<br>Needle width: 0.35 mm<br>Needle pitch: 0.50 mm<br>Angle tip: 20°<br>Thickness: 50 µm

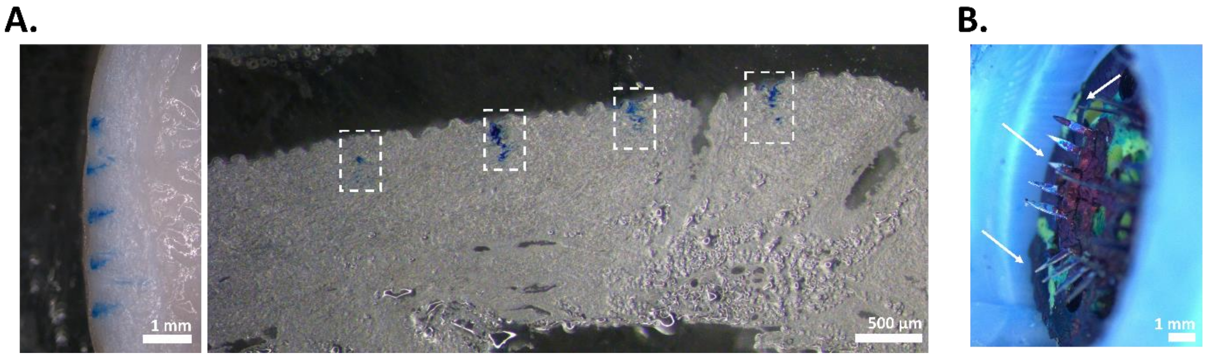

Figure S11. (**A.**) Cross-sectional micrographs of the porcine ear skin after the *ex-vivo* puncturing experiment with a dye-coated circular patch composed of 4 Bl-1 (20 MNs in total), confirming a shallow penetration depth. (**B.**) Circular MN array composed of 4 Bl-4 (20 MNs in total).

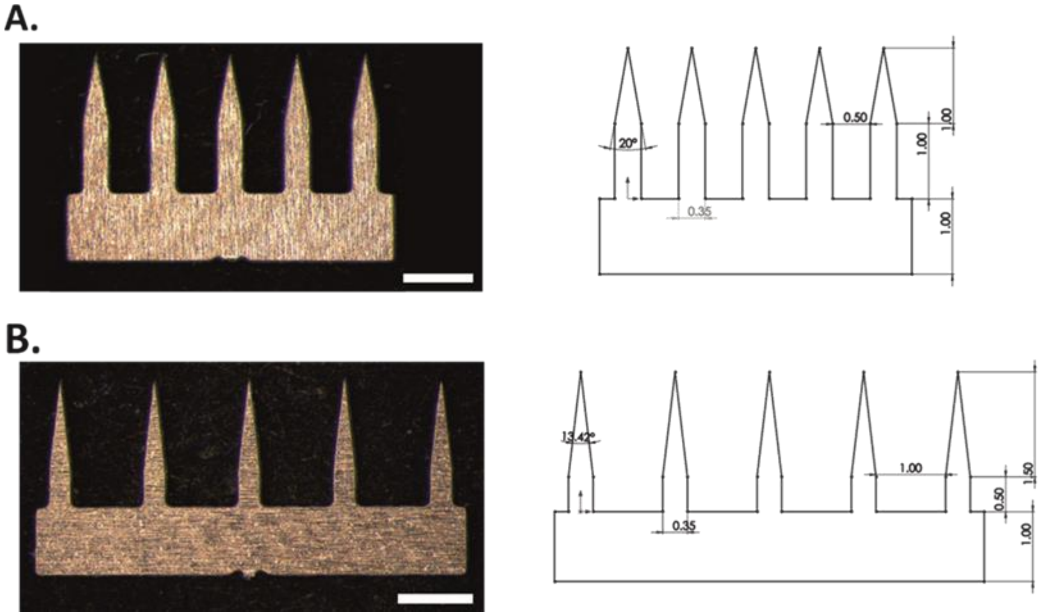

Figure S12. Optical micrographs (left) and sketches (right) of the MN blades used in the initial device development: (**A.**) Bl-2 and (**B.**) Bl-3. Scale bar: 1 mm.

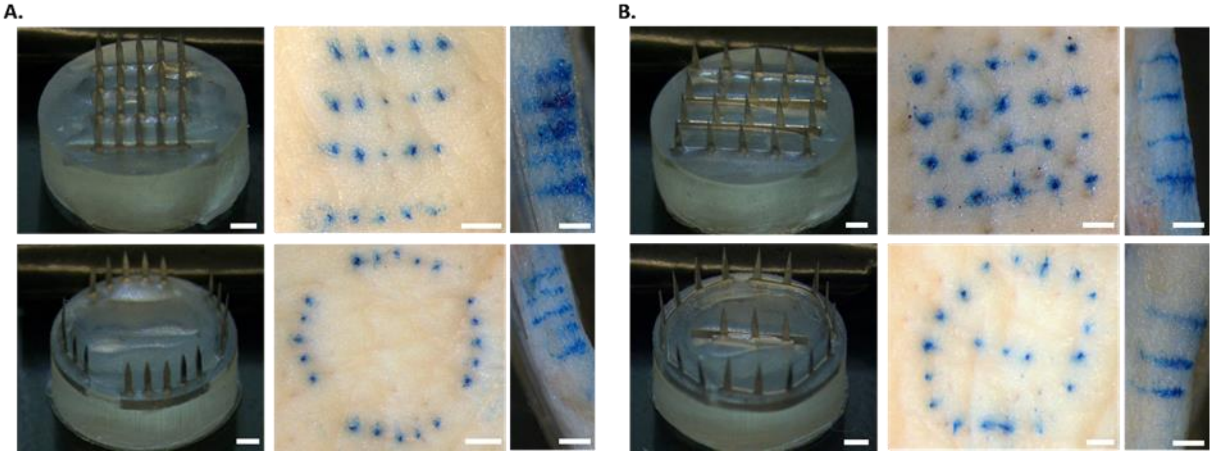

Figure S13. Photographs of the MN patches tested for the initial device development and optical micrographs of the porcine skin (top and cross-sectional view) after the *ex-vivo* puncturing experiment. (**A.**) Rectangular (top) and circular (bottom) MN array composed of Bl-2. (**B.**) Rectangular (top) and circular (bottom) MN array composed of Bl-3. Scale bar: 1 mm.

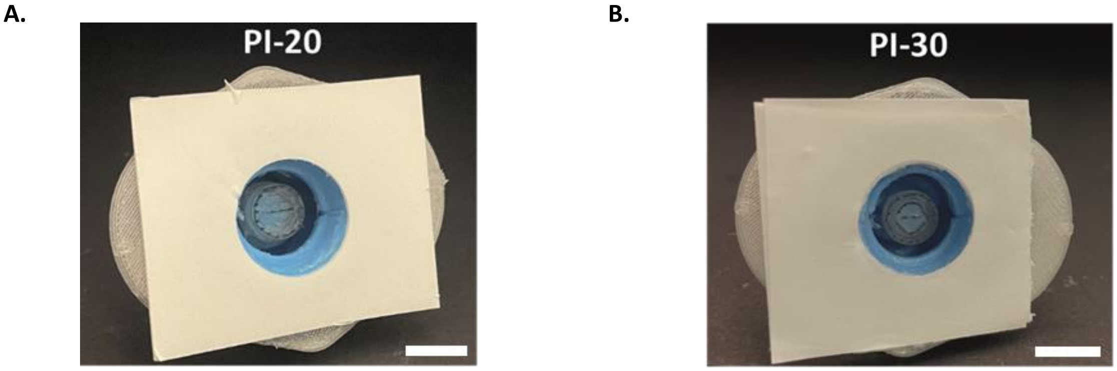

Figure S14. Bottom view photographs of the assembled PI featuring (**A.**) 20 (PI-20) and (**B.**) 30 (PI-30) MNs. Scale bar: 1 cm.

Table S4. Structural characteristics of PI-20 and PI-30.

	**PI-20**	**PI-30**
**Device geometry**	Final Device	Final Device
**Silicone type**	ShA 50	ShA 50
**MN patch geometry**	Circular	Circular
**Blade type**	Bl-3	Bl-3
**Number of MNs**	20 (4 blades)	30 (6 blades)

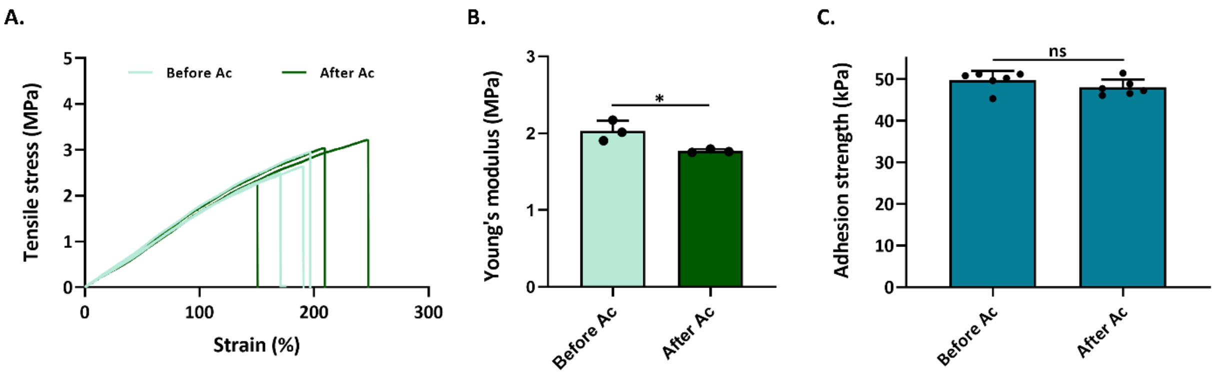

Figure S15. (**A.**) True stress-strain curves and (**B.**) average Young's moduli obtained in a tensile test with cast-molded dog-bone-shaped specimens composed of ShA 50 silicone before (light green) and after (dark green) the autoclaving process. The tensile properties are expressed as mean + SD (n = 3). Statistical significance was calculated by unpaired t-test with a two-tailed distribution and unequal variance with $^{*}P < 0.05$. (**C.**) Adhesion strength of the blood sampling device before and after autoclaving. The study involved manually compressing the devices onto fresh extracted porcine skin and measuring adhesion strength during pull-off at a 0° angle. Data are expressed as mean + SD (*n* = 6). Statistical significance was calculated by unpaired t-test with a two-tailed distribution and unequal variance. (ns: $P > 0.05$).

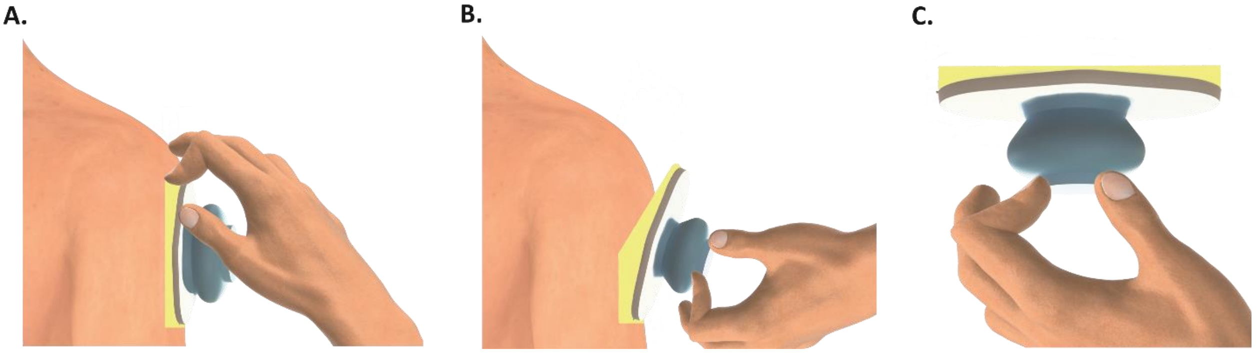

Figure S16. Schematic representation of device removal (**A.**) The prototype can be easily detached from the skin by peeling the tape off from one of its top corners. (**B.**) During removal, the device's knob should be kept facing downward, while the bottom part of the device, in contact with the body, should be kept upward. (**C.**) Correct holding position for the device after removal.

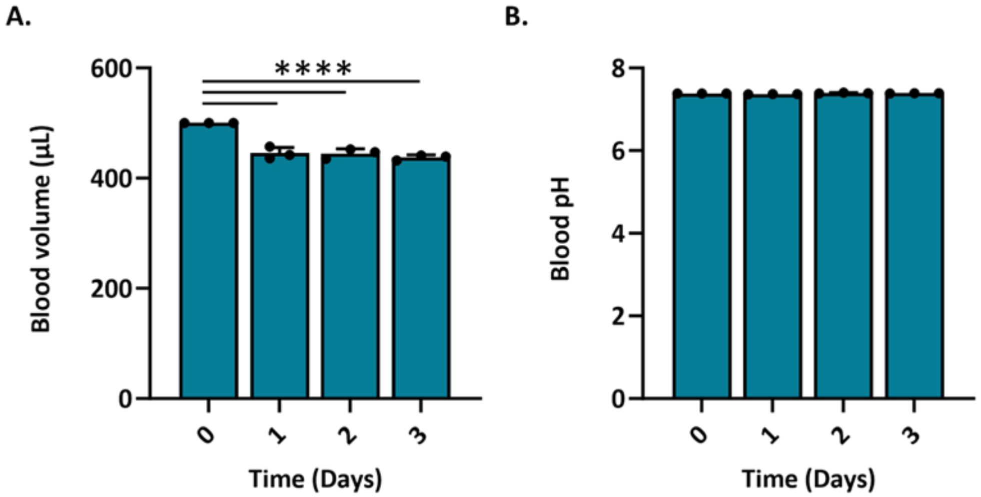

Figure S17. (**A.**) Recovered volume and (**B.**) pH of human whole blood stored within the storage compartment of PI-30 during a 3-day stability study at RT. Specifically, 500 µL of whole human blood (a volume equal to those sampled by the device in-vitro) were loaded into the storage compartment. Subsequently, the device was sealed using the tailored lid and placed inside a sealed bag. At predefined intervals (0, 1, 2, and 3 days), the sealed bag was opened, the lid removed, and the blood volume within the storage compartment was quantified. Data are expressed as mean + SD (*n* = 6). Statistical significance was calculated by two-way analysis of variance (ANOVA) with Tukey's comparison test with ****P < 0.0001. The slight variations in volumes compared to day 0 can be solely attributed to the residual blood remaining within the device (inner walls) and in the pipette tips after blood transfer.

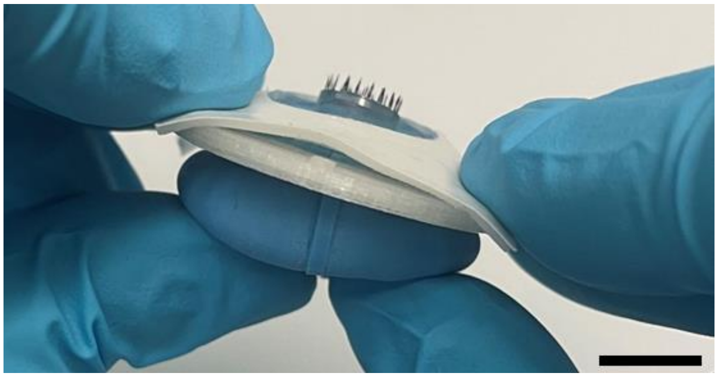

Figure S18. Photograph showing the complete exposure of the MN patch on the correct prototype's compression. Scale bar: 1 cm.

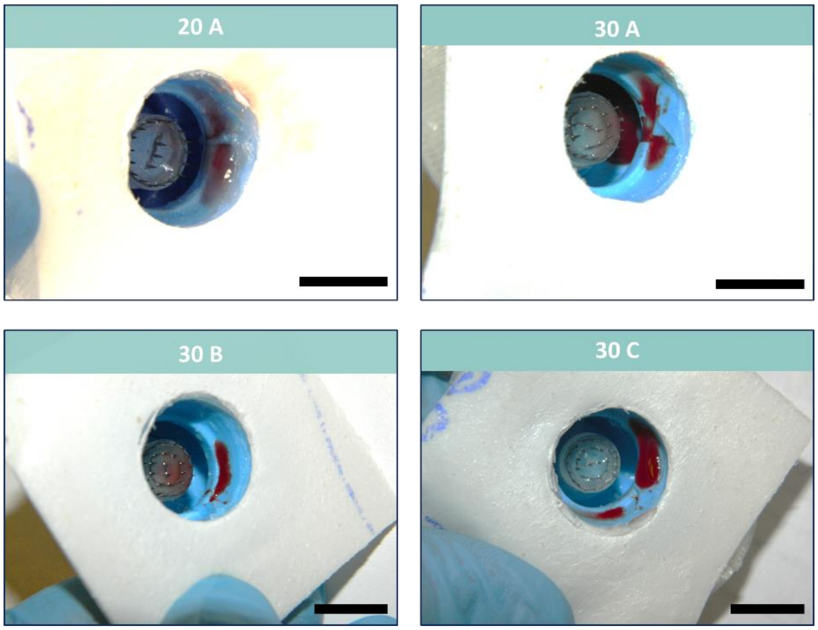

Figure S19. Photographs of PI-20 (20 A) and PI-30 (30A, 30B and 30 C) along with the extracted blood after 10-min application. Pictures 20 A, 30 A and 30 B refer to piglet #1, whilst picture 30 C pertains to piglet #2. Scale bar: 1 cm.

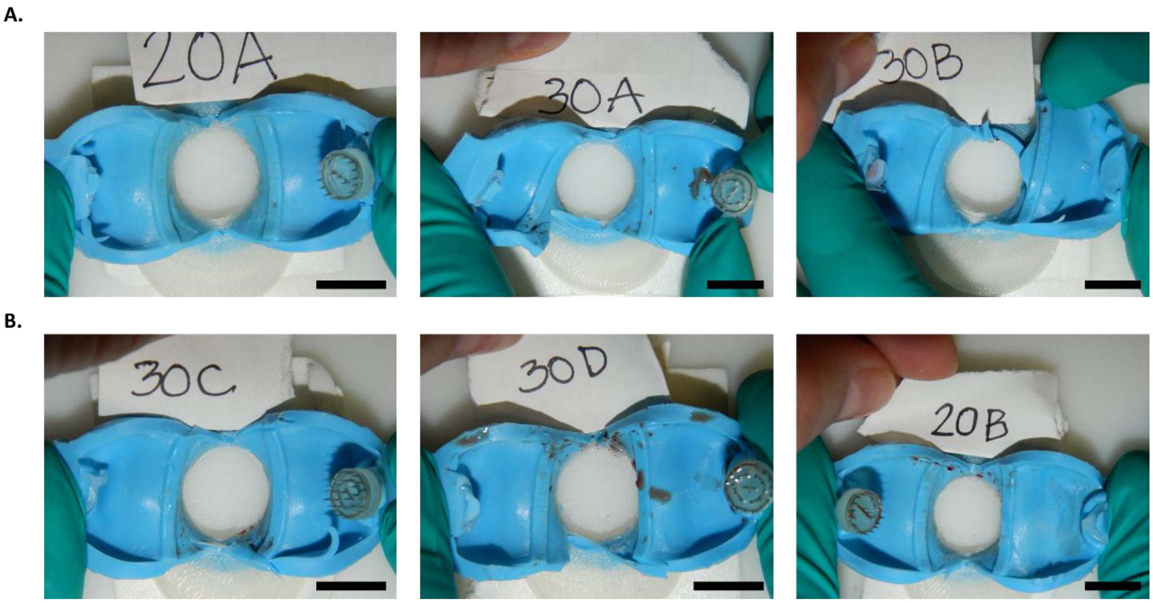

Figure S20. Photographs of all the prototypes tested *in-vivo* after blood transfer into Eppendorf tubes. Devices labeled as 20 A and 20 B refer PI-20, whilst devices labeled as 30 A, 30 B, 30 C and 30 D refer to PI-30. (**A.**) Devices applied on piglet #1. (**B.**) Devices applied on piglet #2. Scale bar: 1 cm.

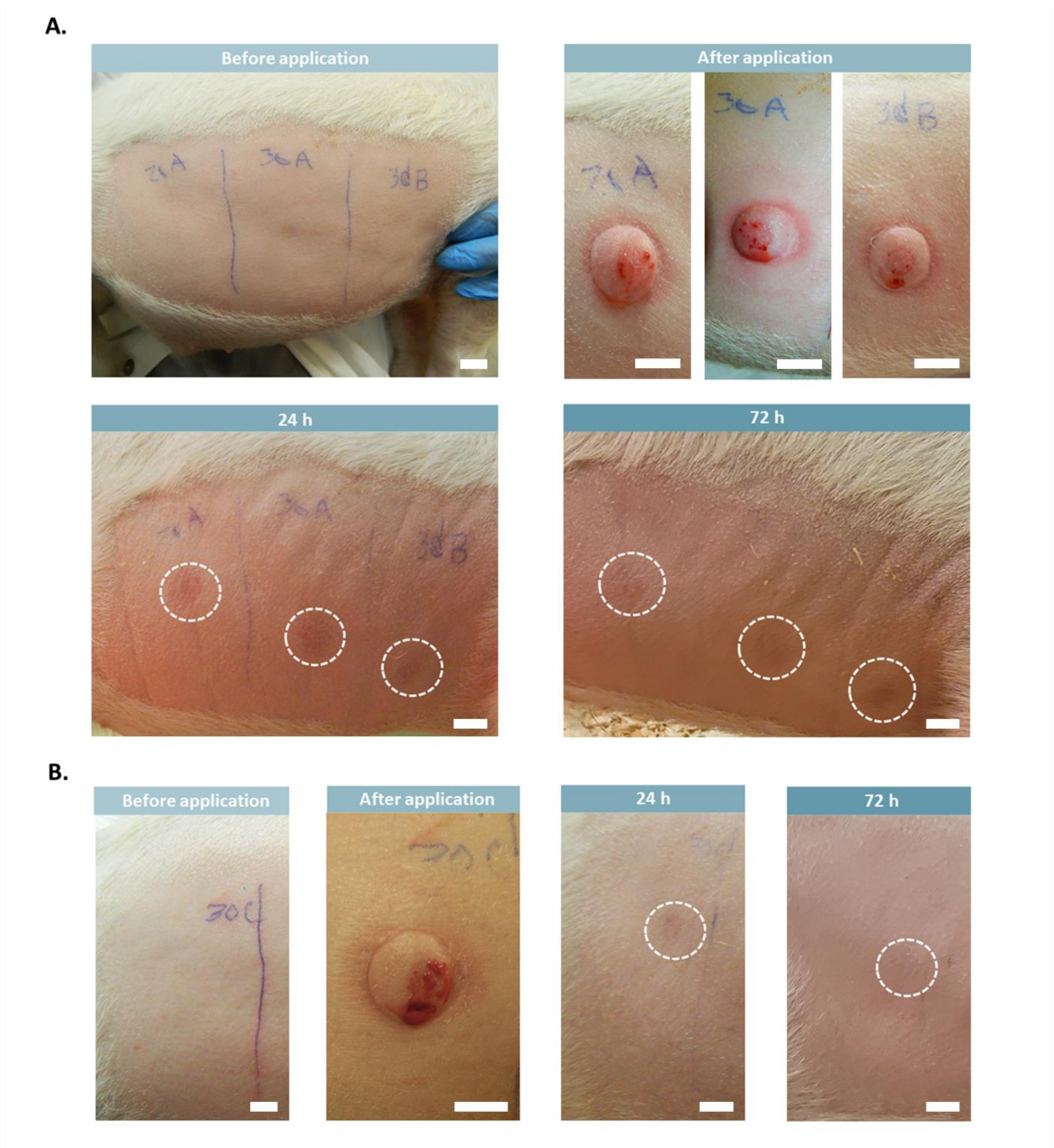

Figure S21. Images of the piglet's skin prior to application, immediately post-application, at 24 h and at 72 h after application. The application site is indicated with a dashed white circle. (**A.**) Photographs of the skin of piglet #1. The labels 20 A, 30 A and 30 B refer to PI-20 (20 A) and PI-30 (30 A and 30 B). (**B.**) Photographs of the skin of piglet #2. The label 30 C refers to PI-30. Scale bar: 1 cm.

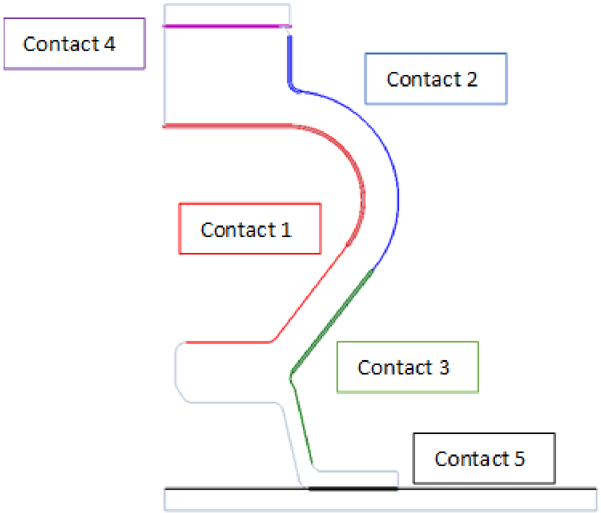

Figure S22. The two-platform approach employed in FEM simulation for device compression, including all the relevant contact conditions (as an example device 3 is shown). The lower platform was designated to maintain a predefined zero deformation, while the upper platform was responsible for the application of the compression force. In each contact pair, the thicker line represents the source term while the thinner line denotes the destination boundary.

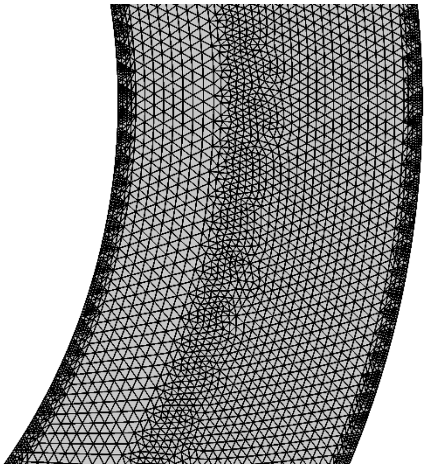

Figure S23. Free triangular mesh employed in FEM simulation for device compression. Here, the meshing of a section of the storage compartment of device 3 is shown. The entire geometry was meshed using the predefined element size "Extremely fine" in COMSOL with a halved maximum element size. Both the inner and outer boundaries of the storage compartment were refined twice. Additionally, the entire geometry was refined twice. The compression platforms were meshed using the predefined "Normal" setting in COMSOL.

Table S5. Convergence study of the mesh for all devices. The maximum element size was doubled for the mesh comparison of each device, resulting in deviations of the average elastic strain energy density of less than 5%. The change in volume compression between the meshes was less than 2%. Therefore, the mesh for each device was assumed to be converging. The increased computational cost here was negligible.

	**Volume (mm^3)**	**Diff. (%)**	**Average strain energy density (J/mm^3)**	**Diff. (%]**	**Maximum strain energy density (J/mm^3)**	**Number of elements**	**Average quality**	**Maximum element size (mm)**
**Device 1**	958.06	0.02	1.9129	2.09	0.70537	19971	0.8161	0.224
	957.9		1.8737		0.35249	40973	0.8516	0.112
**Device 2**	2382.7	0.38	2.0401	0.95	0.23798	23434	0.7795	0.209
	2373.7		2.021		0.19427	60402	0.8331	0.1045
**Device 3**	1528.6	0.89	6.7682	0.69	0.30787	25383	0.7999	0.234
	1515.1		6.722		0.28661	54963	0.8356	0.117
**Device 3**	1527.8	0.89	9.8525	0.70	0.22455	25845	0.7921	0.234
	1514.3		9.7841		0.41459	54963	0.8356	0.117
**Device 3**	1527.8	0.89	20.203	0.70	0.9115	25845	0.7921	0.234
	1514.3		20.062		0.85013	54963	0.8356	0.117
**Device 12**	2108.3	1.23	11.076	3.49	0.56774	107770	0.8576	0.265
	2082.7		10.702		0.57184	100509	0.8643	0.1325
**Device 14**	1737.8	0.51	29.703	3.84	0.65629	33451	0.8541	0.265
	1729		28.604		0.98585	42024	0.8688	0.1325
**Device Final**	1924.9	-0.36	23.922	0.56	3.5231	44130	0.8039	0.25
	1931.9		23.789		3.4858	81965	0.8532	0.125

Table S6. Contact parameters as chosen in the FEM simulations for the contact pairs shown in Figure S19. The penalty method was used for all contacts. Although most penalty factors were consistent across all devices, some were slightly adjusted to enhance convergence. Specifically, contact pair number 2 was set at a very low value to enable a slight phasing of the outer cup boundary with the compression part.

**Contact pair**	**Penalty factor control**	**Penalty factor multiplier**
1	Manual	0.01 (device 14)<br>1 (devices 1 and 12)<br>5 (all the other devices)
2	Manual	0.001 (device 1 and final device)<br>1E-6 (all the other devices)
3	Automatic, soft	1 (devices 1, 12 and 14)<br>0.01 (all the other devices)
4	Automatic	1
5	Automatic, soft	1 (device 14 and final device)<br>0.01 (all the other devices)

A. **B.**

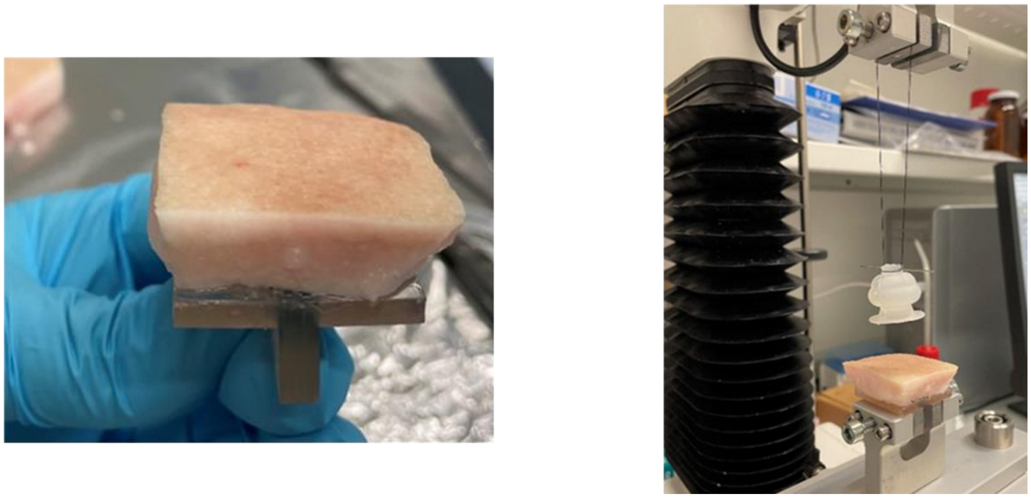

Figure S24. The 0° platform configuration (**A.**) and setup (**B.**) for pull-off force measurements. The device was connected to the pulling clamp using cotton thread and then manually applied to the skin on the 0° platform.

A. **B.**

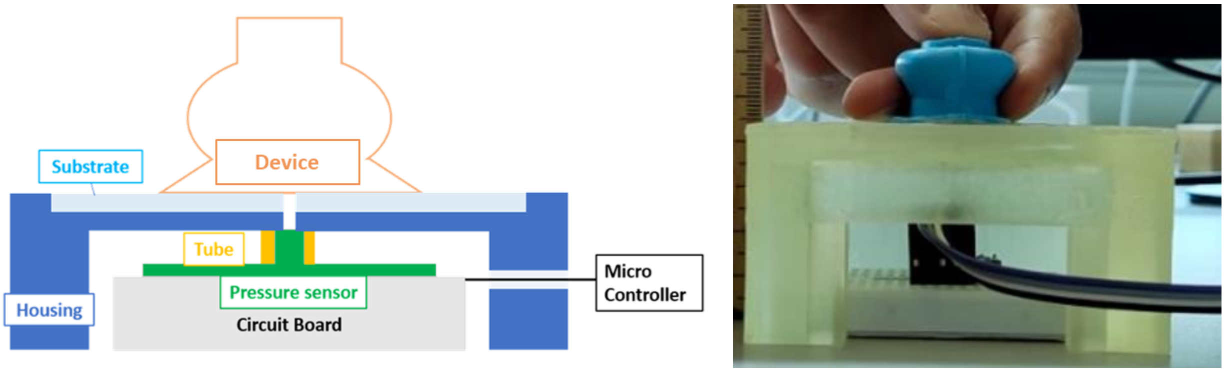

Figure S25. (**A.**) Schematic representation of the pressure setup, consisting of a ShA 33 silicone substrate (mimicking human skin), tubing, pressure sensor, 3D-printed housing, microcontroller and circuit board. To measure the negative pressure generated by the device's compression, devices were manually compressed on the silicone surface. (**B.**) Photograph of the pressure setup during the device application.

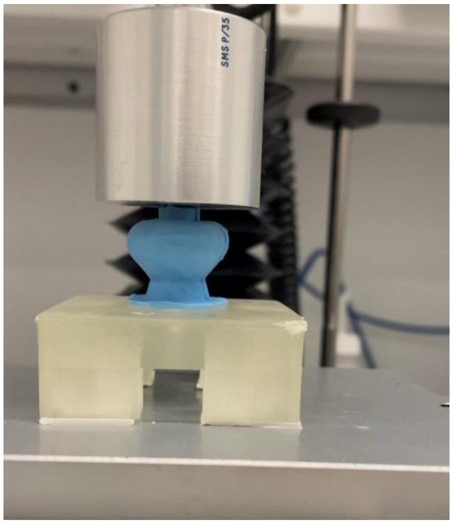

Figure S26. Experimental setup for device displacement measurements. The device was positioned on a 3D-printed platform featuring a central hole and subjected to compression using a texture analyzer at a speed of 1 mm s^{-1}.

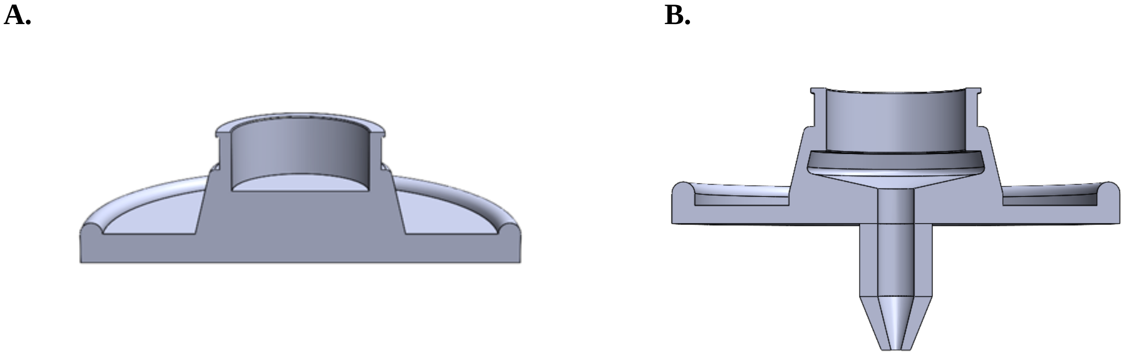

Figure S27. 3D cross-section view of the (**A.**) lid and (**B.**) adapter designed for integration with the final device.
